# Basmisanil, a highly selective GABA_A_-α5 negative allosteric modulator: preclinical pharmacology and demonstration of functional target engagement in man

**DOI:** 10.1038/s41598-021-87307-7

**Published:** 2021-04-08

**Authors:** Joerg F. Hipp, Frederic Knoflach, Robert Comley, Theresa M. Ballard, Michael Honer, Gerhard Trube, Rodolfo Gasser, Eric Prinssen, Tanya L. Wallace, Andreas Rothfuss, Henner Knust, Sian Lennon-Chrimes, Michael Derks, Darren Bentley, Lisa Squassante, Stephane Nave, Jana Nöldeke, Christoph Wandel, Andrew W. Thomas, Maria-Clemencia Hernandez

**Affiliations:** 1 Neuroscience & Rare Diseases (NRD) Discovery and Translational Area, Roche Pharmaceutical Research and Early Development, Roche Innovation Center Basel, Grenzacherstrasse 124, 4070 Basel, Switzerland; 2 Pharmaceutical Sciences, Roche Pharmaceutical Research and Early Development, Roche Innovation Center Basel, Grenzacherstrasse 124, 4070 Basel, Switzerland; 3 Small Molecule Research, Roche Pharmaceutical Research and Early Development, Roche Innovation Center Basel, Grenzacherstrasse 124, 4070 Basel, Switzerland; 4 Pharmaceutical Sciences, Roche Pharma Research and Early Development, Roche Innovation Center Welwyn, Welwyn Garden City, AL7 1TW UK; 5 CNS Research, Roche Bioscience, Palo Alto, USA; 6 Present Address: AbbVie Inc, North Chicago, IL USA; 7 Present Address: Galwyn (UK) Ltd, Dartmouth, Devon UK; 8 Present Address: BlackThorn Therapeutics, 780 Brannan Street, San Francisco, CA 94103 USA; 9 Present Address: Helvetica Capital AG, Zurich, Switzerland

**Keywords:** Drug discovery, Neuroscience, Biomarkers, Medical research

## Abstract

GABA_A_-α5 subunit-containing receptors have been shown to play a key modulatory role in cognition and represent a promising drug target for cognitive dysfunction, as well as other disorders. Here we report on the preclinical and early clinical profile of a novel GABA_A_-α5 selective negative allosteric modulator (NAM), basmisanil, which progressed into Phase II trials for intellectual disability in Down syndrome and cognitive impairment associated with schizophrenia. Preclinical pharmacology studies showed that basmisanil is the most selective GABA_A_-α5 receptor NAM described so far. Basmisanil bound to recombinant human GABA_A_-α5 receptors with 5 nM affinity and more than 90-fold selectivity versus α1, α2, and α3 subunit-containing receptors. Moreover, basmisanil inhibited GABA-induced currents at GABA_A_-α5 yet had little or no effect at the other receptor subtypes. An in vivo occupancy study in rats showed dose-dependent target engagement and was utilized to establish the plasma exposure to receptor occupancy relationship. At estimated receptor occupancies between 30 and 65% basmisanil attenuated diazepam-induced spatial learning impairment in rats (Morris water maze), improved executive function in non-human primates (object retrieval), without showing anxiogenic or proconvulsant effects in rats. During the Phase I open-label studies, basmisanil showed good safety and tolerability in healthy volunteers at maximum GABA_A_-α5 receptor occupancy as confirmed by PET analysis with the tracer [^11^C]-Ro 15-4513. An exploratory EEG study provided evidence for functional activity of basmisanil in human brain. Therefore, these preclinical and early clinical studies show that basmisanil has an ideal profile to investigate potential clinical benefits of GABA_A_-α5 receptor negative modulation.

## Introduction

GABA_A_ receptors are a family of ligand-gated ion channels which respond to the major inhibitory neurotransmitter, GABA. There are 19 genes encoding for GABA_A_ receptor subunits that assemble as pentamers, with the most common stoichiometry of two α, two β, and one γ subunit^1^. GABA has two binding sites at the interface of the α and β subunits. Many compounds in clinical use as anxiolytics, sedatives, hypnotics or antiepileptics bind to the allosteric benzodiazepine (BZD) binding site which is formed by one of the α subunits (α1, α2, α3 or α5) and usually the γ2 subunit. These compounds are known as positive allosteric modulators (PAMs) since they have no effect alone, but increase the activity of GABA_A_ receptors in the presence of GABA^2,3^. In contrast, negative allosteric modulators (NAMs) at the BZD-binding site decrease the activity of GABA_A_ receptors. Non-selective GABA_A_ receptor NAMs have so far only been tested in experiments on animal behavior and in very few exploratory human studies. They showed cognition enhancing properties; however, further clinical development of these non-selective compounds was prevented by anxiogenic or proconvulsive side effects^4–9^.

Both genetic and pharmacological studies have demonstrated that GABA_A_-α5 subunit-containing receptors play an important modulatory role in learning and memory processes, in line with their preferential expression in the hippocampus and cortical regions^10^. Several compounds have been described to possess selectivity for the α5 containing receptors such as, α5IA^11^, MRK-016^12^, PWZ-029^13^, RO4938581^14^, ONO-8290580^15^ and in preclinical studies have demonstrated cognitive enhancement without the anxiogenic or proconvulsant side effects associated with non-selective NAMs^4,6^.

GABA_A-_α5 NAMs hold promise as potential treatments for multiple indications, particularly associated with cognitive impairment. Initially, it was proposed that selective GABA_A_-α5 NAMs may be beneficial for Alzheimer’s Disease and mild cognitive impairment^16^. Since then, further preclinical studies suggest that GABA_A_-α5 NAMs may attenuate cognitive impairment associated with Down syndrome (DS)^17,18^, cognitive impairment associated with schizophrenia (CIAS) as shown by improvements in preclinical models of NMDA dysfunction^19–21^ and cognitive impairment after anesthesia^22^. In addition, studies in mice and rats have shown that after ischemic stroke there is increased GABA_A_-α5 mediated tonic inhibition in the peri-infarct area and that reduction of GABA_A_-α5 activity enhances functional recovery after stroke^23–25^. Recently, GABA_A_-α5 NAMs have also been shown to exert rapid antidepressant-like effects in mice^26,27^.

**Figure 1:**
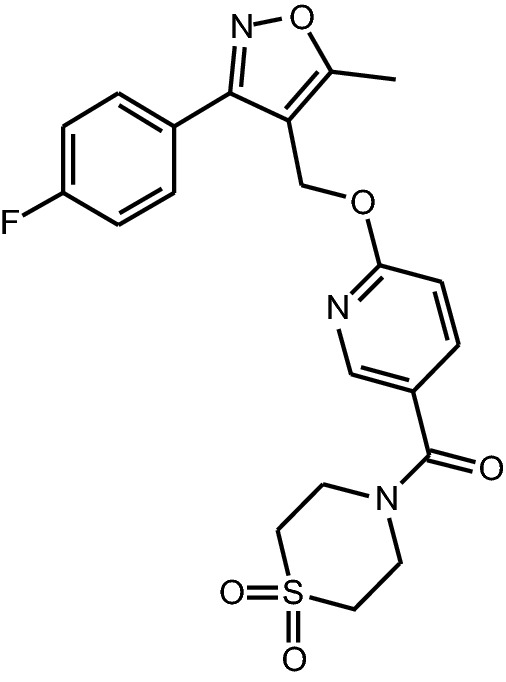
Chemical structure of basmisanil: RO5186582; RG1662; C21H20FN3O5S; molecular weight 445.469; 1,1-Dioxo-1,6-thiomorpholin-4-yl)-{6-[3-(4-fluoro-phenyl)-5-methyl-isoxazol-4-ylmethoxy]-pyridin-3-yl}-methanone.

However, as of today we are not aware of a selective and safe GABA_A_-α5 NAM undergoing clinical development. α5IA and MRK-016 were tested in clinical trials, but their development was stopped because of renal toxicity in preclinical species^28^ and poor tolerability in healthy elderly humans as well as variable pharmacokinetics, respectively^12,29^. Interestingly, in an experimental medicine study, α5IA reversed an alcohol-induced impairment in word list recall^30^. PWZ-029 and ONO 8290580 have not been reported in clinical studies more likely due to unfavorable pharmacokinetic and/or safety profiles. Our previous compound, RO4938581, had to be abandoned because of strong CYP1A2 autoinduction properties in rats leading to reduced plasma exposure on chronic dosing^31^, but our continued search for GABA_A_-α5 selective NAMs without safety liabilities led to basmisanil (Fig. 1). Basmisanil was discovered in a medicinal chemistry effort starting from the results of a 56,265 small molecular-weight compound library high-throughput screen based on [^3^H]-flumazenil competition-binding followed by electrophysiological experiments on cloned GABA_A_ receptor subtypes to determine the functional activity of the initial hits.

Basmisanil has already been assessed in Phase II clinical studies for intellectual disability in DS (https://clinicaltrials.gov/ct2/show/NCT02024789) and CIAS (https://clinicaltrials.gov/ct2/show/NCT02953639). A trial was started in stroke recovery but was terminated due to the low enrolment of participants (https://clinicaltrials.gov/ct2/show/NCT02928393). Reporting on the outcome of these Phase II studies is beyond the scope of this publication.

The aim of this study was to provide an assessment of the preclinical and early clinical pharmacological profile of basmisanil. The preclinical studies included compound selectivity and estimated range of receptor occupancies required for in vivo efficacy, which guided clinical doses for Phase II trials. The early clinical data established the relationship between plasma exposure and receptor occupancy (PET), provided the first evidence of functional CNS activity (EEG; tested at high receptor occupancy only) and the safety profile in healthy human volunteers.

## Results

### Basmisanil exhibits high affinity and selectivity for human GABA_A_-α5 receptors

**Figure 2:**
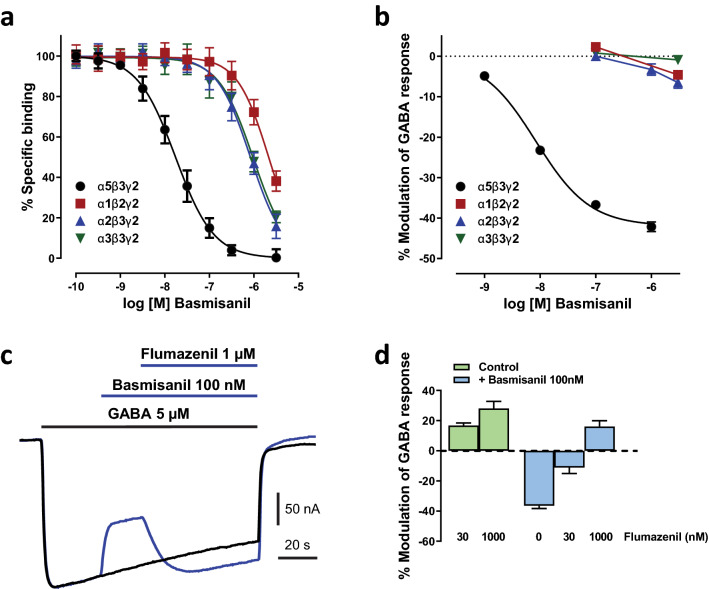
Basmisanil is a potent and highly selective GABA_A_-α5 receptor NAM. Data in the graphs are shown as mean (symbols) ± SEM (error bars). Error bars smaller than the symbol size are not shown. (**a**) Concentration–response curves of basmisanil in [^3^H]-flumazenil competition-binding assays to membranes expressing different human recombinant GABA_A_ receptor subtypes (n = 7–9 per concentration). (**b**) Concentration–response curves of the effects of basmisanil on ion current induced by an EC_10_ of GABA in Xenopus oocytes (n = 7–8 per concentration). (**c,d**) Flumazenil antagonism of basmisanil effects. (**c**) Example recording of ion current from a voltage-clamped Xenopus oocyte expressing human GABA_A_-α5. Two current traces are superimposed. The control response (black) was evoked by a GABA application in the absence of any modulators, as indicated by the black horizontal bar. During the test response (blue), after a delay, basmisanil and then flumazenil (blue horizontal bars) were added to GABA. (**d**) Bar graph showing flumazenil antagonism of the basmisanil effect. Green bars: flumazenil alone (30 nM: n = 5, 1000 nM: n = 11). Blue bars: flumazenil in presence of basmisanil (100 nM basmisanil: n = 16; basmisanil + flumazenil 30 nM: n = 6; basmisanil + flumazenil 1000 nM: n = 10).

As shown in Fig. 2a, basmisanil bound with high affinity (Ki = 5 ± 1 nM) and more than 90-fold selectivity to membranes containing GABA_A_ α5β3γ2 receptors compared to HEK293 cells expressing the human GABA_A_ receptor subunits α1 (Ki = 1031 ± 54 nM), α2 (Ki = 458 ± 27 nM) or α3 (Ki = 510 ± 21 nM) in conjunction with the β3 and γ2 subunits. The high affinity of basmisanil to human GABA_A_ α5β3γ2 receptors was confirmed in a [^3^H]-Ro-15 4513 displacement assay yielding a similar Ki of 7.6 ± 0.3 nM. Comparable affinity and selectivity were found for rat GABA_A_ receptors expressed in HEK293 cells (data not shown). Basmisanil binding to human GABA_A_ α5β3γ2 showed > 600-fold selectivity compared to human α4β3γ2 and α6β3γ2, i.e., no binding at > 3 µM (data not shown).

Further experiments were conducted by Eurofins CEREP to assess the binding of basmisanil (10 µM) to 78 other receptors, transporters and ion channels (Supplementary Table S2). The compound showed at least 2000-fold selectivity for the GABA_A_ α5β3γ2 receptor over all the targets tested with exception of the sigma receptor, where it produced 58% displacement of the label, [^3^H]-1,3 di-ortho-tolylguanidine [^3^H]-DTG.

### Basmisanil exhibits functional selectivity for human GABA_A_-α5 receptors

To assess the intrinsic efficacy of basmisanil, two electrode voltage-clamp experiments were performed in *Xenopus* oocytes. GABA was applied repetitively at a concentration evoking approximately 10% of the maximal response (8 µM for α1β2γ2, 6 µM for α2β3γ2, 10 µM for α3β3γ2 and 5 µM for α5β3γ2). The maximum possible negative allosteric modulation of the GABA responses was determined by using the non-selective NAM, methyl beta-carboline-3-carboxylate (β-CCM). Basmisanil showed a highly selective inhibition of GABA_A_-α5 (Fig. 2b). At the highest concentration tested (1 µM), basmisanil reduced the GABA-evoked current by up to 42 ± 1%, which is slightly less effective than β-CCM (52% ± 6%, data not shown). The IC_50_ value and Hill slope calculated from the concentration–response curve (Fig. 2b) were 8 nM and 0.9, respectively (n = 7). Basmisanil was only weakly active at GABA_A_ receptors containing other α subunits (< 10% inhibition at 3000 nM) (Fig. 2b). In contrast, β-CCM inhibited the other GABA_A_ receptors to a similar degree than that observed at GABA_A_-α5, being − 50% ± 1%, 42% ± 3% and − 51% ± 3% at α1β2γ2, α2β3γ2 and α3β3γ2 GABA_A_ receptors, respectively (not shown).

We also looked for the functional competition between basmisanil and flumazenil, a non-selective weak PAM at cloned GABA_A_ receptors (Fig. 2c,d). Flumazenil concentration-dependently (30 and 1000 nM) enhanced the GABA-induced current in α5-expressing oocytes up to 28%. This effect is much weaker than that of midazolam, a full PAM, causing 145 ± 9% increase at 1000 nM (n = 12, not shown). Basmisanil administered alone, decreased the GABA-induced current and this effect was blocked by co-treatment with flumazenil (Fig. 2d)**.**

### Dose-dependent GABA_A_-α5 receptor occupancy by basmisanil in rat brain

**Figure 3:**
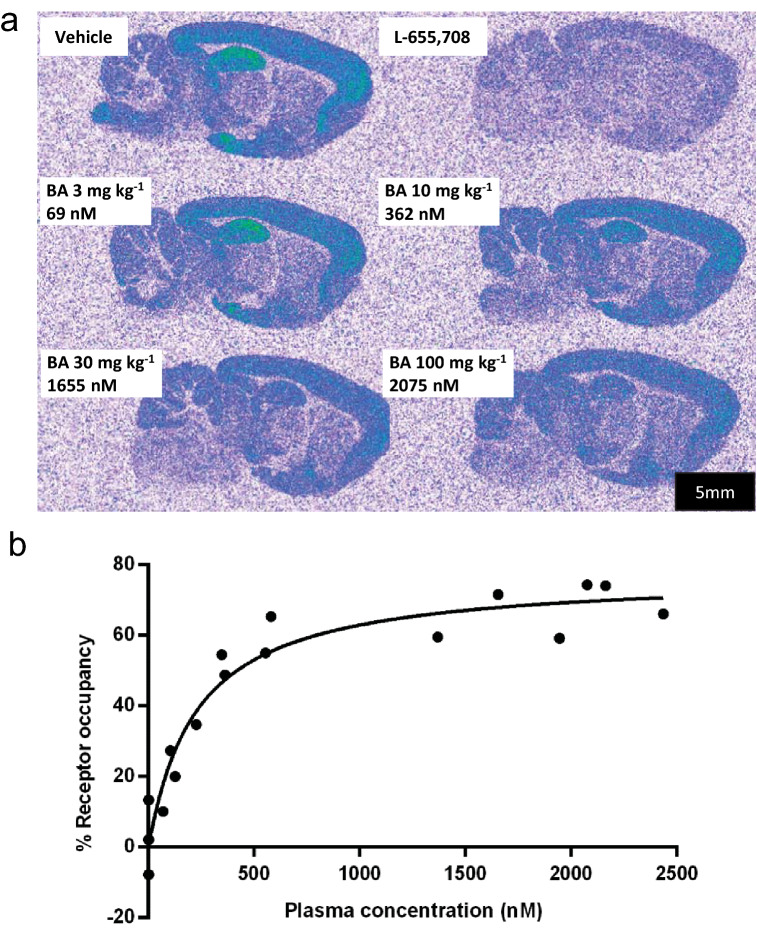
Dose-dependent GABA_A_-α5 receptor occupancy by basmisanil in rat brain. Dose-dependent blockade of [^3^H]-Ro 15-4513 binding by basmisanil determined 60 min after p.o. administration of basmisanil and 15 min after i.v. injection of [^3^H]-Ro 15-4513. (**a**) Sagittal brain sections of female Sprague–Dawley rats pretreated with vehicle (n = 4), L-655,708 (10 mg/kg; n = 4) or increasing doses of basmisanil at 3 mg/kg (n = 4), 10 mg/kg (n = 4), 30 mg/kg (n = 4) and 100 mg/kg (n = 2). (**b**) Quantitative autoradiography signals in the hippocampus were plotted against individual plasma concentrations of basmisanil. The curve was fitted to the experimental results using the Hill equation (Eq. (1) with Hill coefficient D = 1).

[^3^H]-Ro 15-4513 (ethyl 8-azido-6-oxo-5-(tritritiomethyl)-4H-imidazo[1,5-a][1,4]benzodiazepine-3-carboxylate) was employed as a radioligand to estimate in vivo GABA_A_-α5 occupancy of basmisanil in rats. This radioligand has high but not complete selectivity for GABA_A_ α5β3γ2 receptors^32,33^ which complicates the quantification of drug occupancy at this receptor subtype. We discovered during our programme that the rat exhibited large gender dimorphism in intrinsic clearance of basmisanil, as often observed due to higher metabolic rates in male rats. Hence female rats were chosen for the receptor occupancy study, as they showed higher systemic exposure (5- to 11-fold higher) compared to male rats. Ex vivo autoradiographical analysis after intravenous injection of [^3^H]-Ro 15-4513 revealed a binding pattern that is in good agreement with the known distribution of GABA_A_-α5 receptors^34,35^. Strongest binding under baseline conditions was observed in brain regions known to contain high densities of GABA_A_-α5 receptors, such as the hippocampus and the frontal cortex, whereas regions containing less GABA_A_-α5 such as the striatum and the cerebellum showed lower densities of [^3^H]-Ro 15-4513 binding (Fig. 3a). Due to the limited selectivity of Ro 15-4513 for the GABA_A_-α5 receptor subtype, binding to non-α5 GABA_A_ receptor subtypes and ‘standard’ non-specific binding of the radioligand was estimated by a cohort of rats which were pretreated with the α5-selective ligand L-655,708 (10 mg/kg i.p.) (Fig. 3a)^36^. To obtain the specific binding, the radioactivity of L-655,708-treated animals was subtracted from the radioactivity in the corresponding brain region of vehicle-treated animals. Densities of specific [^3^H]-Ro 15-4513 binding sites in vehicle-treated animals were 304 ± 15, 192 ± 15, 25 ± 5 and 38 ± 4 fmol/mg protein in the hippocampus, frontal cortex, striatum and cerebellum, respectively.

Pre-treatment with basmisanil (3–100 mg/kg p.o.) decreased the binding of [^3^H]-Ro 15-4513 in a dose-dependent manner (Fig. 3a). The highest dose (100 mg/kg) reduced specific binding in the hippocampus by 70 ± 6% (blockade by 10 mg/kg L-655,708 was defined as 100% blockade). Binding data were further analysed by plotting receptor occupancy against the plasma concentration of basmisanil of each individual (Fig. 3b). Fitting the data by the Hill equation (i.e., Eq. (1)) resulted in a plasma level of 544 ± 125 ng/mL (171 ± 16 nM free plasma concentration) for producing half-maximal receptor occupancy (EC_50_).

### Basmisanil improved cognition in rats and non-human primates

Our previous compound, RO4938581, was shown to have cognition enhancing effects in rodents in the Morris water maze and non-human primates in the object retrieval test^37^. The water maze was selected to assess the effects of basmisanil on a hippocampal-mediated test of spatial learning and memory^37^. Object retrieval was selected to assess the effects of basmisanil on a task of prefrontal cortical-mediated cognition which involves planning and response inhibition, i.e., executive function^38^.

#### Basmisanil attenuated diazepam-induced spatial learning impairment in rats

In the water maze, rats are challenged to find a hidden platform position when placed in a large pool filled with opaque water. Diazepam impairs spatial learning in naïve rats in this paradigm and it has been proposed that non-selective GABA_A_ receptor PAMs may induce memory impairment by modulating hippocampal function^39,40^. Diazepam significantly increased the latency and pathlength to find the platform position across six trials and induced a small but significant increase in swim speed (Supplementary Fig. S2). Basmisanil did not significantly reduce the diazepam-induced increase in latency and pathlength and had no effect on swim speed (Supplementary Fig. S2).

**Figure 4:**
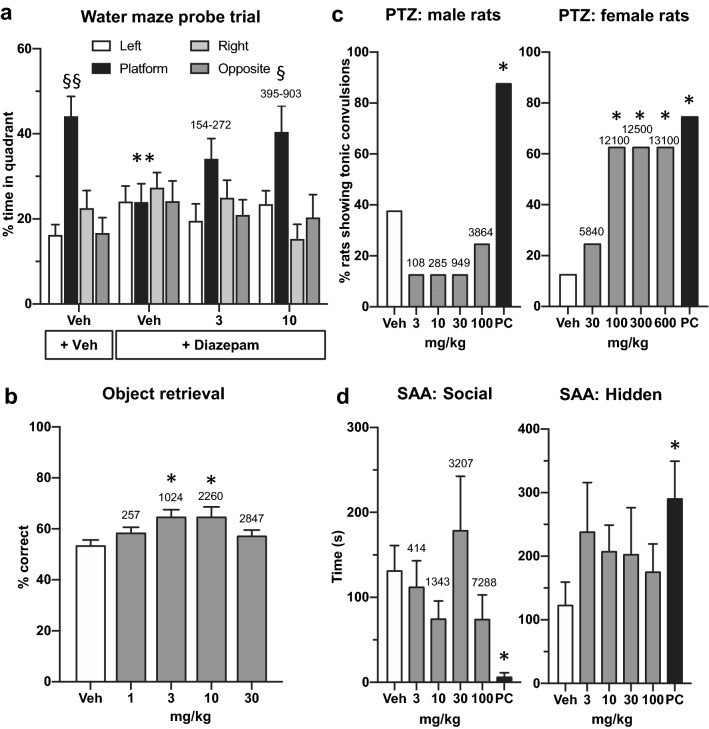
Basmisanil improved cognition in rodents and non-human primates at plasma concentrations which did not show anxiogenic or proconvulsant effects. (**a**) Basmisanil reversed the diazepam-induced spatial learning impairment of rats in the Morris water maze. Basmisanil (3 and 10 mg/kg p.o. as indicated below the abscissa) and diazepam (6 mg/kg i.p.) were administered to male Lister Hooded rats 30 min before the test. Data are presented as mean ± SEM (n = 10 per dose group). Statistics: *Significant difference between vehicle and diazepam-treated groups on percent time in platform quadrant (unpaired t-test); § significant difference between percent time spent in platform quadrant versus left, right and opposite quadrants (post hoc Newman-Keuls test). Total plasma concentrations (ng/mL) for each dose are shown above each bar and were collected in parallel to the experiment (n = 4 per dose). The range shows the concentrations at 30 and 100 min post-administration which are equivalent to the beginning and end of testing. (**b**) Basmisanil improved executive function in adult cynomolgus macaques. The effect of basmisanil administered at 1, 3, 10 and 30 mg/kg p.o. on percent correct (first reaches) during difficult trials of the object retrieval task. Data are presented as mean ± SEM (n = 12/dose; within-subjects design). Statistics: *Significant difference versus vehicle-treated group (post hoc Dunnett’s test). Total plasma concentrations (ng/mL) for each dose are shown above each bar. Samples were collected at 180 min for the same three subjects. (**c**) Effect of basmisanil and the positive control (PC), FG7142, on the percent of male or female rats with tonic convulsions following administration of a threshold dose of PTZ (n = 8 per treatment group). *Significant difference versus vehicle group (Pearson Chi-Square test). Plasma samples were collected either immediately following tonic convulsions or 30 min following PTZ, i.e., approximately 60–90 min (males) or 180–210 min (females) post administration of basmisanil (n = 8 per dose). (**d**) Effect of basmisanil administered p.o. 1 h prior to testing and the positive control (PC), Ro 19-4603 administered p.o. 30 min prior to testing, in the social approach avoidance test in male F-344 rats (n = 10 per treatment group). Data are expressed as mean ± SEM. *Significant difference versus vehicle group (unpaired t-test). Total plasma concentrations (ng/mL) for each dose are shown above each bar. Plasma samples were collected immediately after testing i.e., approximately 75 min post administration of basmisanil (n = 4 per dose).

The platform was removed before the seventh trial. The vehicle group demonstrated spatial learning of the platform position, as indicated by the significant (F(3,27) = 9, p < 0.001) increase in percent time spent in the platform quadrant compared to left (p < 0.001), right (p < 0.001) and opposite (p < 0.001), quadrants (Fig. 4a). Diazepam significantly disrupted learning of the new platform position since subjects spent an equivalent amount of time in each quadrant during the probe trial (F(3,27) = 0.1, p = 0.9). Moreover, there was a significant difference between vehicle and diazepam-treated groups (p < 0.01) in the percent time spent in the platform quadrant. Basmisanil at 10 mg/kg p.o. significantly (F(3,27) = 4, p = 0.01) attenuated the diazepam-induced deficit as revealed by an increase in percent time spent in the previous platform quadrant compared to left (p < 0.05), right (p < 0.05) and opposite (p < 0.05) quadrants (Fig. 4a).

Plasma concentrations of basmisanil as determined in parallel dose groups were dose- and time-dependent (Fig. 4a) and reached a maximal level of 903 ± 13 ng/mL (379 ± 5 nM free plasma) 30 min after the administration of basmisanil at 10 mg/kg. Using Fig. 3b and assuming that the asymptotic maximum of the curve actually corresponds to 100% occupancy of GABA_A_-α5 receptors, occupancy during the water maze experiment was estimated to be between 45 and 65%.

#### Basmisanil improved percent correct in the object retrieval task in adult cynomolgus macaques

Young adult cynomolgus macaques are pre-trained to this task so that performance of difficult trials is around 50% correct responses. Basmisanil significantly improved the percentage of correct first reaches during difficult trials of the object retrieval task at the 3 and 10 mg/kg doses (F(4,36) = 5.26, p = 0.0019; Fig. 4b). Basmisanil exhibited an inverted U-shaped dose response in this paradigm with the 1 and 30 mg/kg doses producing no marked improvement on performance. The total plasma exposure increased with dose (Fig. 2b). Active plasma concentrations were 1024 and 2260 ng/mL (156 and 345 nM free plasma) which corresponded to about 30 and 50% estimated receptor occupancy.

In summary, these studies confirm that GABA_A_-α5 NAMs enhance cognition in rodents and non-human primates in both hippocampal and prefrontal cortex mediated tasks. Basmisanil showed an overlap in the receptor occupancy range required for efficacy across both tests.

### Pre-clinical de-risking studies for anxiety and convulsions

A potential risk of non-selective GABA_A_ NAMs is to induce epileptic activity and anxiety^4–9^. Therefore, we tested basmisanil for proconvulsant effects in the pentylenetetrazole (PTZ) assay and anxiogenic effects in the social approach avoidance (SAA). We included doses which were higher than those tested in the cognition assays.

#### Lack of proconvulsant activity of basmisanil at therapeutically relevant, but not at supra-therapeutic concentrations

The potential for proconvulsant activity of basmisanil was evaluated in the PTZ test in Wistar rats. Initially, male rats were assessed and single doses of basmisanil up to 100 mg/kg (total plasma concentration of 3864 ng/mL; 1622 nM free plasma concentration) did not exert proconvulsant activity in the PTZ test (Fig. 4c). In order to explore higher doses of basmisanil, we exploited the gender difference in plasma exposure (described above) and tested female rats. Basmisanil at 30 mg/kg in female rats, (total plasma concentration of 5840 ng/mL; 2452 nM free plasma concentration; Fig. 4c) did not exert proconvulsant activity (Fig. 4c). The receptor occupancies estimated at these plasma concentrations are approximately 60–70% for α5 receptors, which are the maximum observed in the in vivo binding experiments described above. In female rats at a dose of 100 mg/kg (total plasma concentration of 12,100 ng/mL; 5079 nM free plasma concentration; Fig. 4c) five out of eight rats exhibited tonic convulsions (p < 0.05) following administration of a threshold dose of PTZ. The two higher doses produced identical effects (p < 0.05) and had similar plasma concentrations to 100 mg/kg (Fig. 4c). The positive control, FG7142 (*N*-Methyl-9*H*-pyrido[5,4-*b*]indole-3-carboxamide) a non-selective GABA_A_ receptor NAM, significantly (p < 0.05) increased the number of rats showing tonic convulsions, as expected (Fig. 4c).

#### Basmisanil did not have an anxiogenic-like effect in the social approach avoidance test in rats

In the SAA experiment, during a 10 min session a F-344 Fischer rat can freely move between a non-social compartment (with hidden and protected areas) and a social compartment containing a large, unfamiliar stimulus rat (Sprague–Dawley) behind a perforated wall (with proximal and distal areas to the unfamiliar rat). The preference for the hidden area or for the social compartment has been shown to reflect the anxiety state of the test animal and the test is suitable for assessing potential anxiolytic-like and anxiogenic-like effects of compounds^41^. Basmisanil did not significantly reduce the time spent in the social compartment (F(4,45) = 1.4, p = 0.3) or increase the time spent in the hidden area (F(4,45) = 0.6, p = 0.7) up to 100 mg/kg (Fig. 4d). Plasma concentrations between 414 and 7288 ng/mL (174 to 3059 nM free plasma concentrations) were found (Fig. 4d) with estimated receptor occupancies of 50 to 77%. As expected^41^, Ro 19-4603, the non-selective GABA_A_ NAM used as a positive control, significantly decreased the time in the social compartment (p < 0.001) and increased the time in the hidden area (p < 0.05; Fig. 4d).

In summary, these studies confirm that a GABA_A_-α5 NAM does not have an anxiogenic-like or proconvulsant effect in rodents at therapeutic plasma concentrations.

### Dose-dependent GABA_A_-α5 receptor occupancy by basmisanil in human brain of healthy volunteers

**Figure 5:**
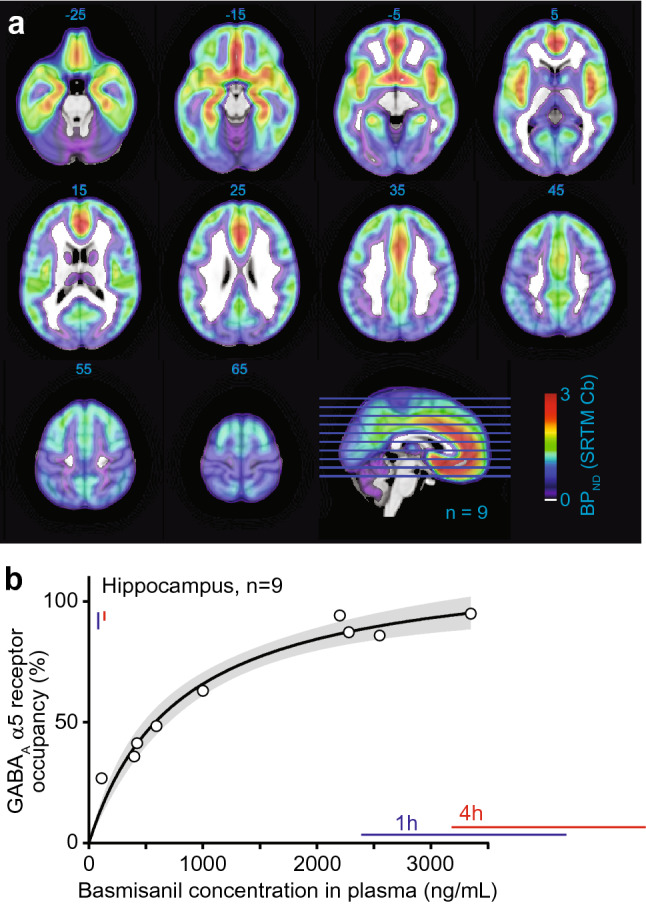
Dose-dependent GABA_A_-α5 receptor occupancy by basmisanil in human brain. (**a**) Representative [^11^C]-Ro 15-4513 PET images in healthy human subjects showing the GABA_A_-α5 receptor distribution. *BP*_*ND*_ binding potential, *SRTM* simplified reference tissue model, *Cb* cerebellum. Numbers for each slice indicate the axial slice location in MNI coordinates. Blue and red bars indicate plasma exposure and receptor occupancy ranges (mean ± standard deviation) at time-points where EEG was recorded (1 h and 4 h post morning dose after 14 days of 240 mg basmisanil dosing twice daily). (**b**) Relation between basmisanil concentration in plasma and receptor occupancy derived from nine subjects. Emax model fit (E = Emax × C/(EC_50_ + C)), gray band indicates 95% confidence interval determined by the profile likelihood method. Blue and red bars indicate mean ± SD of blood plasma concentrations in the EEG experiment at 1 h and 4 h post morning dose at day 14, respectively.

The degree of receptor occupancy by basmisanil was determined in healthy volunteers using PET and [^11^C]-Ro 15-4513. PET images of the brain at baseline displayed a spatially structured signal consistent with previously published [^11^C]-Ro 15-4513 PET data and the known distribution of GABA_A_-α5 subunit-containing receptors, with the highest signal in the hippocampus, nucleus accumbens, amygdala and cortical regions^42^; Fig. 5a). Following administration of basmisanil, heterogeneity in both summed PET images and tissue time activity curves was reduced relative to baseline scans, indicating blocking of [^11^C]-Ro 15-4513 GABA_A_-α5 receptor binding by basmisanil. A dose-dependent decrease in [^11^C]-Ro 15-4513 binding in all regions was observed after administration of basmisanil. The relationship between receptor occupancy and plasma concentrations could be described by a simple Emax model, suggesting a direct relationship i.e., no significant delay in receptor occupancy compared to plasma concentration, with an EC_50_ of approximately 541 ng/mL total plasma concentration corresponding to 67 nM free plasma concentration (Fig. 5b). The calculated GABA_A_-α5 receptor occupancy at the highest dose of 1000 mg basmisanil reached 94% in the hippocampus (Supplementary Table S5).

### Electrophysiological signature of basmisanil in healthy volunteers

Twelve healthy volunteers underwent resting state EEG assessments at baseline, after an acute dose of midazolam (a short-acting non-selective GABA_A_ PAM), and after 14 days of sub-chronic treatment with basmisanil. Treatment with basmisanil (240 mg twice daily) led to high occupancy of GABA_A_-α5 receptors of > 90% (plasma exposure at 1 h 3284 ± 887.9 ng/mL and 4 h post morning dose 4030 ± 842.3 ng/mL; receptor occupancy at 1 h 92 ± 3% and 4 h post morning dose 94 ± 1.5%; exposure and occupancy ranges during EEG assessments are depicted in Fig. 5b).

**Figure 6:**
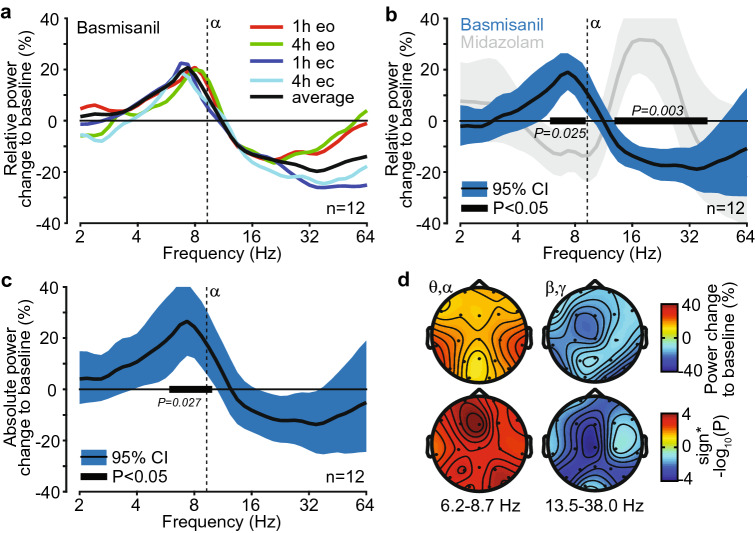
Electrophysiological signature of basmisanil in healthy volunteers. (**a**) Relative power change of EEG signals averaged across electrodes in response to basmisanil, separately for eyes open and eyes closed conditions at 1 and 4 h post morning dose after 14 days of 240 mg basmisanil treatment twice daily. (**b**) Relative power change of EEG signals averaged across electrodes in response to basmisanil (average of eyes open and eyes closed conditions, 1 h and 4 h post morning dose after 14 days of 240 mg basmisanil treatment twice daily) and in response to midazolam (grey: eyes open and eyes closed, 1 h post single dose of 2 mg). Black bars indicate significant effects for basmisanil (cluster randomization test). (**c**) Absolute power change of EEG signals averaged across all electrodes in response to basmisanil (average of eyes open and eyes closed conditions, 1 and 4 h post morning dose after 14 days of 240 mg basmisanil treatment twice daily). Black bar indicates significant effect (cluster randomization test). (**d**) Spatial distribution of power changes and p-values (derived from t-tests) for the two significant clusters identified for basmisanil (black bars in (**b**)). Top panel: power changes; bottom panel: p-values; left: low-frequency cluster (6.2–8.7 Hz); right: high-frequency cluster (13.5–38.0 Hz). *CI* confidence interval (uncorrected for multiple testing across frequencies). See Supplementary Fig. S5 for additional EEG analyses and Fig. 5 for basmisanil exposure and receptor occupancy ranges during EEG assessments.

The relative spectral power change of basmisanil from baseline was similar across time-points investigated (1 h and 4 h post morning dose) and for eyes open and eyes closed conditions (Fig. 6a). We therefore averaged all four recordings for the analysis reported in the main text (see Supplementary Fig. S5c–f for separate analyses, which provide very similar results). Basmisanil significantly increased relative power in the theta- to low alpha frequency range (6.2–8.7 Hz; Fig. 6b) by 19.1 ± 3.3% compared to pre-treatment baseline (p = 0.025). The peak frequency of the effect was 7.3 ± 0.4 Hz, which was below the mean alpha peak frequency of 9.3 Hz. Furthermore, basmisanil significantly decreased relative power in the beta- to low gamma frequency range (13.5–38.0 Hz; peak frequency of 34.9 ± 3.13 Hz) by 18.9 ± 4.24% compared to the pre-treatment baseline (p = 0.003). Absolute power showed a similar spectral response, while only the low-frequency peak survived multiple-comparison correction (Fig. 6c).

The spectral pattern induced by basmisanil was qualitatively opposite to that of midazolam, characterized by a decrease of relative power at low frequencies in the high theta to low alpha bands (8.2 ± 4.12 Hz, − 14.4 ± 3.73%) and an increase at higher frequencies in the beta band (18.4 ± 6.06 Hz, 32.2 ± 7.04%) (Fig. 6b, gray band; Supplementary Fig. S5g). However, the peak frequency of the beta to gamma band effect was significantly higher (p = 0.024) for basmisanil compared to midazolam (no significant difference for low-frequency peaks). Furthermore, the spatial topography of the beta to gamma band effects significantly differed (p = 0.0023) between basmisanil and midazolam (within/between drug cross-subject correlation r = 0.128/0.003; Fig. 6d, Supplementary Fig. S5h), likely reflecting the difference in expression pattern of targeted receptors.

### Safety in healthy human volunteers in the PET and EEG studies

Basmisanil was safe and well tolerated in the healthy subjects evaluated in the PET and EEG studies. There were no severe adverse events, discontinuations or temporary discontinuations due to adverse events, in any cohort.

## Discussion

Our preclinical studies showed that the in vitro pharmacological profile of basmisanil translates into efficacy in cognition paradigms together with an adequate safety profile at estimated GABA_A_-α5 receptor occupancy of around 30–65%. Our clinical studies demonstrated direct target engagement using PET and our first EEG study provided evidence for functional target engagement of basmisanil in humans.

Basmisanil is a highly selective GABA_A_-α5 NAM. Basmisanil bound to the BZD binding site of recombinant human GABA_A_-α5 with 5 nM affinity and more than 90-fold selectivity versus α1, α2, and α3 subunit-containing receptors, as demonstrated by our flumazenil competition binding assays. Basmisanil also showed functional selectivity, since it inhibited GABA-induced current in cells expressing GABA_A_-α5 yet had little or no effect at the other receptor subtypes. The potencies for basmisanil in the binding and electrophysiology experiments fitted well together, being in the nano molar range. By combining the highly selective preference in affinity and efficacy at GABA_A_-α5 versus GABA_A_ α1, α2, and α3 subunit-containing receptors, basmisanil represents the most selective and potent GABA_A_-α5 NAM in clinical development described so far (see Supplementary Table S4).

Our in-vivo binding experiments using [^3^H]-Ro 15-4513 as a radiolabel for GABA_A_-α5 receptors along with an ex vivo autoradiography readout resulted in a dose-dependent blockade of radiotracer binding by increasing doses of basmisanil. A plasma level of 544 ± 125 ng/mL basmisanil (171 ± 16 nM free plasma concentration) produced half-maximal receptor occupancy (EC_50_). The PK-occupancy curve for basmisanil approached a maximum at 77%. Thus, 23% of the [^3^H]-Ro 15-4513 binding sites could not be occupied by basmisanil. This can be explained by the limited selectivity of the radiolabel for GABA_A_-α5 containing receptors combined with the relative higher abundance of α1- and α2-containing receptors and the quantification method used in this preclinical study. For Ro 15-4513 published affinity ratios α1/α5 or α2/α5 range between 11 and 15^32,36,43^. In the hippocampus, α5-containing receptors were found to account for 16–26% of all GABA_A_ receptors^44,45^. Consequently, the partial selectivity of [^3^H]-Ro 15-4513 for the GABA_A_-α5 receptor subtype has some implications for the distinction of α5 versus non-α5 contributions of radioligand binding. In our experimental approach, ‘off-target’ radioligand binding to non-α5 GABA_A_ receptor subtypes together with ‘classical’ non-specific binding was estimated by a cohort of rats which were pretreated with the α5-selective ligand L-655,708 (10 mg/kg i.p.). The corresponding binding level of [^3^H]-Ro 15-4513 in the brains of this cohort was defined as 100% occupancy. L-655,708 binds with high affinity to the GABA_A_-α5 receptor subtype (K_i_ ≈ 1 nM) and has 30 to 70-fold selectivity over other GABA_A_-receptor subtypes. However, a high dose of 10 mg/kg L-655,708 will also occupy non-α5 receptor subtypes to some extent. Basmisanil has more than 200-fold selectivity for the GABA_A_-α5 receptor subtype versus the major non-α5 receptor subtype α1β3γ2. Thus, even at a high dose of 100 mg/mg, basmisanil only occupies GABA_A_-α5 receptors and cannot block the remaining 23% of [^3^H]-Ro 15-4513 binding sites attributable to non-α5 receptor subtypes. In similar experiments with our previous α5-selective NAM, RO4938581, up to 90% of the binding sites of [^3^H]-Ro 15-4513 in the hippocampus were occupied by the compound^14^. The apparent higher receptor occupancy of RO4938581 compared to basmisanil is due to the lower binding selectivity of RO4938581, which was about 40-fold versus α1- and α2-containing receptors^14^. Overall, the in vivo binding experiment presented in this study demonstrated dose-dependent target engagement of basmisanil in rats and confirmed the very high selectivity for GABA_A_-α5 receptors in vivo.

Preclinical experiments suggest pro-cognitive effects of basmisanil. In the spatial learning and memory test, acute administration of basmisanil at 10 mg/kg (estimated receptor occupancy between 45 and 65%) significantly attenuated the diazepam-induced impairment. Reversal of diazepam impairment has been proposed to be mediated via reduced activation of GABA_A_-α5 receptors^14^. In NHPs basmisanil improved performance in the object retrieval task at estimated GABA_A_-α5 receptor occupancy between 30 and 50%, which is similar to the rodent study. However, there was an inverted U-curve which was not observed in the rodent study. The lack of effect of higher doses was not due to lack of exposure, since plasma concentrations increased dose-dependently. A possible explanation may be that in the rodent study basmisanil was assessed in a pharmacologically-impaired model and so had to reverse an induced cognitive deficit. In non-impaired rodents GABA_A_-α5 NAMs, including basmisanil and RO4938581, have not shown enhancement of cognitive performance (data not shown). In contrast the NHPs were healthy, unimpaired adults which performed at lower accuracy due to task difficulty. In this healthy condition, strongly decreasing GABA_A_-α5 activity may not be beneficial for cognition. In summary, basmisanil exhibited cognition enhancing properties in pharmacologically-induced spatial learning impairment in rats and in an executive function task in NHPs.

Basmisanil was found to be safe (non-convulsive, non-anxiogenic) in preclinical experiments and Phase I clinical studies. Importantly, at plasma concentrations shown to improve cognition with estimated receptor occupancy of 30–65% and higher, basmisanil lacked anxiogenic and proconvulsant potential, underlining the critical importance of subtype selectivity in binding affinity and functional efficacy (Fig. 4c). Moreover, sensorimotor gating was not impaired by basmisanil at receptor occupancy of 40–50% (Supplementary Fig. S3); although higher doses were not tested. In the PTZ test, basmisanil exhibited proconvulsant activity only at supra-therapeutic high plasma exposure, which may be due to binding at other α subtypes and potentiation of PTZ activity by allosteric conformational changes in the receptor. It has been suggested that application of the PTZ test to assess the proconvulsant activity of drugs that antagonize GABA either directly or indirectly may lead to false positive conclusions and over-estimate the pro-convulsive risk of the molecule in question^46^. Preclinical good laboratory practice (GLP) toxicology and safety pharmacology studies have shown no convulsions and no EEG alterations following daily oral administration of basmisanil at supra-therapeutic plasma exposures for up to 26 weeks in rats and 39 weeks in dogs (see Supplementary Fig. S4). The discrepancy between the PTZ test and the GLP toxicology studies may be related to synergistic activity through PTZ in the GABAergic system.

To confirm absence of pro-convulsive risk in humans at therapeutic exposures, EEG monitoring was included in several Phase I studies covering high plasma exposures of basmisanil (see Supplementary Clinical Information). Maximum plasma concentration values observed in the multiple ascending dose (MAD) study (approximately 8250 ng/mL, n = 6) and drug-drug interaction (DDI) study (approximately 14,500 ng/mL, n = 12) exceeded those needed for full receptor occupancy at trough by approximately six-fold, showing that we have a good safety window for Phase II trials. EEG readings did not reveal signals of pro-convulsive effects, such as epileptiform abnormalities, mirroring the high receptor selectivity shown in vitro and in line with the lack of clinical signs and convulsion symptoms in GLP toxicology studies in dogs at exposures up to 4590 ng/mL.

Basmisanil engages with the target (PET) and modulates neuronal activity (EEG). PET imaging data, obtained in vivo in healthy human volunteers using the [^11^C]-labelled version of Ro 15-4513, indicated that basmisanil enters the brain and blocks the binding of the PET tracer in a dose dependent manner. The relationship between receptor occupancy and basmisanil plasma concentration could be described by a simple Emax model suggesting a direct relationship, i.e., no significant delay in receptor occupancy compared to plasma concentration, with an EC_50_ of approximately 541 ng/mL total plasma concentration, corresponding to 67 nM free plasma concentration. This EC_50_ is in good agreement with the half-maximal receptor occupancy observed in the rat study (EC_50_ = 171 ± 16 nM free plasma concentration). The similarity of these values across species is also notable in view of the different experimental approaches and quantification methods which were employed to assess occupancy in rats and humans, respectively. A similarly good translatability of PK-occupancy relationship across species and methodological approaches has been previously demonstrated for the α5-selective GABA_A_ receptor ligands α5IA and MRK-016^12,47^. Comparison of the observed in vivo EC_50_ of basmisanil in rats and humans with in vitro affinity values determined using recombinant α5β3γ2 receptors revealed a 24-fold shift in affinity in rat while only a ninefold affinity shift was observed for the human receptor. Comparable shift factors have been observed for another α5-selective GABA_A_ receptor ligand^12^. Understanding the drug concentration-occupancy relationship allowed us to select dosing regimens for the Phase II clinical trials (DS, CIAS, stroke recovery) to adequately cover a specific target occupancy.

The exploratory EEG assessment in healthy volunteers, revealed that negative modulation of GABA_A_-α5 receptors leads to an increase in lower frequency EEG power (theta to alpha frequency range) and a decrease in higher frequency EEG power (beta to gamma frequency range). These results demonstrate that basmisanil has an effect on brain function and suggest EEG as a pharmacodynamic readout for future clinical studies.

Non-selective GABA_A_ PAMs, i.e. BZDs, are known to induce a characteristic EEG signature with an increase in beta-band activity which is an established pharmacodynamic marker, along with a decrease of activity at lower frequencies^48–50^. The EEG response to midazolam analyzed here well recapitulates this. Non-selective GABA_A_ NAMs (e.g., DMCM, Ro 19-4603) have been shown to reduce beta-band activity in rodents^50^. Furthermore, genetic links between beta-band activity and single nucleotide polymorphisms (SNPs) in GABA_A_ receptor genes^51,52^ and for rare genetic conditions (Dup15q syndrome, Angelman syndrome) involving copy number variations including GABA_A_ genes (*GABRB3, GABRA5, GABRG3*)^53,54^, have been reported. GABA_A_ receptor-related changes in beta-band activity are thought to reflect modulation of recurrent excitatory-inhibitory feedback loops in cortical tissue^55,56^, but are not understood in detail. Thus, the EEG effects of basmisanil are well in line in terms of frequency bands affected and directionality of the change with prior work on GABA_A_ receptors.

The EEG study has limitations, which should be considered. The number of subjects was low (n = 12), there was no placebo condition (only a baseline recordings), and only a clinical EEG with 19 electrodes was used, which makes it difficult to clean high-frequency artifacts to study gamma band activity^57^. Only a high dose (> 90% receptor occupancy) in sub-acute treatment regime (following 14 days of basmisanil dosing) was tested. Furthermore, the effect in the beta frequency range was not significant for absolute power when correcting for multiple testing across frequencies. Future studies need to confirm these findings, identify the pharmacokinetic/pharmacodynamic relationship and understand if the findings also generalize to acute and long-term treatment with basmisanil.

Finally, we would like to conclude with a brief overview of our Phase II trials with basmisanil and our future plans. Our preclinical and early clinical data show that basmisanil has an ideal profile to investigate the potential clinical benefits of GABA_A_-α5 receptor negative modulation. Basmisanil has been investigated for its potential to improve intellectual disabilities associated with DS in two separate trials in adolescents/adults (12–30 years) and children (6–11 years). The former trial was negative and so the latter trial was terminated before completion. There are several possible reasons for the negative outcome such as: translation of the animal model to the human condition, the negative modulatory effect at GABA_A_-α5 receptors was not sufficient for efficacy in this population, the clinical scales were not appropriate to detect drug effects. Alternatively, it is possible that efficacy can only be achieved if treatment starts during early brain development in childhood. The Phase II trial in CIAS was completed and the results are currently being analysed. Another Phase II study to investigate the efficacy of basmisanil in stroke recovery was considered but then abandoned due to recruitment issues. With the analysis of these Phase II trials we are starting to learn more about the effects of this compound in different disorders, and we plan to share this information in forthcoming manuscripts. In addition, we are considering to explore the effects of basmisanil in genetically-driven disorders where there is strong evidence for a role of GABA_A_-α5 receptors, such as the neurodevelopmental disorder Dup15q syndrome^54^.

## Methods

### Materials

Basmisanil was synthesized at F. Hoffmann-La Roche AG (Basel, Switzerland) as described in WO2013/057123 A1^58^. GABA_A_ receptor modulators, i.e., diazepam, midazolam, flumazenil (Ro 15-1788), Ro 19-4603, L-655,708, FG7142, pentylenetetrazole (PTZ) and the radioligands [^3^H]-flumazenil ([^3^H]-Ro 15-1788) and [^3^H]-Ro 15-4513 were also synthesized at F. Hoffmann-La Roche Ltd.

### In vitro studies

Basmisanil was prepared as a 10 mM stock solution in DMSO.

### Radioligand binding assays in HEK293 membrane preparations expressing cloned human GABA_A_ receptors

#### Plasmids and recombinant cell expression

The cDNAs encoding different human GABA_A_ receptor subunits (α1-6, β2-3, γ2) were subcloned into the polylinker of the pcDNA3.1 vector (Invitrogene, USA) by standard techniques for transiently transfecting human embryonic kidney (HEK)293-EBNA cells or for in vitro RNA synthesis.

HEK293-EBNA cells adapted to grow in suspension were transiently transfected with the plasmids containing the desired GABA_A_ receptor subunit cDNAs at α, β, γ, 1:1:2 ratio using XtremeGENE Q2 (Cat. No. 03045595001, Roche Applied Science, RAS, Rotkreuz, Switzerland) as previously described^59^. GABA_A_ α1β2γ2, α2β3γ2, α3β3γ2, α4β3γ2, α5β3γ2 and α6β3γ2 receptor subtypes were expressed in this system. 48 h post transfection, the cells were harvested and washed three times with cold PBS and frozen at − 80 °C.

#### Membrane preparation and radioligand binding assays

Membrane preparations of HEK293 cells expressing the different GABA_A_ receptor subtypes and [^3^H]-flumazenil binding assays were performed as described previously^14^. The inhibition of 1 nM [^3^H]-flumazenil binding by basmisanil was measured in membranes expressing human GABA_A_ α1β2γ2 (n = 9), α2β3γ2 (n = 9), α3β3γ2 (n = 7) and α5β3γ2 (n = 8) receptor subtypes. Non-specific binding was determined in the presence of 10 μM diazepam. The percentage inhibition of [^3^H]-flumazenil binding, the IC_50_ and the Ki values were calculated using ActivityBase (IDBS, Guildford, Surrey, UK) as described below.

The affinity of basmisanil for GABA_A_ receptors containing α4 and α6 subunits was measured using [^3^H]-Ro 15-4513 competition-binding experiments. Membranes were incubated with 1 nM of [^3^H]-Ro 15-4513 and ten concentrations of basmisanil. Nonspecific binding was measured in the presence of 10 μM Ro 15-4513. IC_50_ values were derived from the inhibition curve and Ki values were calculated as described below.

#### Binding affinity

The relation between specific binding and modulator concentration was fitted by the equation:

1 $$ {\text{y }} = {\text{ A }} + \, \left( {{\text{B }} - {\text{ A}}} \right)/\left[ {{ 1 } + \, \left( {{\text{C }}/{\text{ x}}} \right)^{{\text{D}}} } \right], $$

with y = % specific binding, A = minimum y, B = maximum y, C = IC_50_, D = slope (Hill coefficient) and x = concentration of basmisanil. From the estimated value of IC_50_ we calculated the affinity constant (K_i_) using the Cheng–Prusoff equation, K_i_ = IC_50_/(1 + (L/K_d_), where L is the concentration (1 nM) and K_d_ is the affinity constant of the radiolabel, [^3^H]-flumazenil. The previously determined values of K_d_ are 1.14 nM, 1.59 nM, 1.75 nM and 0.46 nM for α1, α2, α3 and α5 containing receptors, respectively. For Ro 15-4513, the concentration was also 1 nM and the Kd values were 1.01 nM and 1.83 nM for α4 and α6 containing receptors, respectively.

### Selectivity screening

Binding of basmisanil (10 µM) to 78 other receptors, transporters and channels was tested by Eurofins Cerep SA (Celle l’Evescault, France) following the methods described on the website, www.eurofinsdiscoveryservices.com.

### Voltage-clamp of ***Xenopus laevis*** oocytes expressing cloned human GABA_A_ receptors

#### Cloning and preparation of RNA for microinjection into *Xenopus laevis* oocytes

The cDNAs encoding different human GABA_A_ receptor subunits were subcloned into the polylinker of the pcDNA3.1 vector (Invitrogene, ThermoFischer Scientific, Waltham, MA, USA) by standard techniques. The constructs were linearized at the 3′ end of the corresponding receptor subunit cDNA with the appropriate restriction enzyme keeping the polymerase promoter site upstream of the sequence to be transcribed (see Supplementary Table S1). Capped and poly(A) tailed cRNA transcripts were synthesized from linearized plasmids encoding the desired protein by using the mMessage mMachine T7 ultra kit (Ambion, ThermoFischer Scientific) according to the recommendation of the manufacturer. The synthesized RNA was purified by using the MEGAclear (Ambion) spin columns. cRNA concentrations were calculated with the Nanodrop 8000 and visualized on 1.5% agarose gels. The RNAs are stored at − 80 °C.

#### Microinjection of oocytes and electrical recording

Freshly prepared oocytes of maturation stage VI were bought from Ecocyte Bioscience (Castrop-Rauxel, Germany) and, after overnight delivery, plated into 96-well plates (Greiner no. 651101) for microinjection on a ROBOOCYTE R16 system (Multichannel Systems MCS GmbH, Reutlingen, Germany). Each oocyte was injected with roughly 50 nL of RNA solution. The solution for expressing receptors composed of the human α1, β2 and γ2 subunits contained 3 pg/nL of α1 and β2 RNA and 15 pg/nL of γ2 RNA. The solution for expressing human α2β3γ2 receptors contained 2.5 pg/nL of α2 and β3 RNA, 5 pg/nL of γ2 RNA and 10 pg/nL of GABARAP RNA. The solution for expressing human α3β3γ2 receptors contained 1.3 pg/nL of α3 and β3 RNA and 6.5 pg/nL of γ2 RNA. The solution for expressing human α5β3γ2 receptors contained 1.3 pg/nL of α5 and β3 RNA and 13 pg/nL of γ2 RNA. GABARAP was added to the α2β3γ2 subunit mixture since the expression of γ2 would be low otherwise, as indicated by a weak effect of β-CCM^60^. After the microinjection the oocytes were kept at 20 °C temperature until the electrophysiological experiment. Experiments were done 3 to 5 days later on the ROBOOCYTE instrument which employs the two-microelectrode voltage-clamp technique for membrane voltage control and current measurement. Glass microelectrodes were filled with a mixture of KCl (1 M) and K-acetate (1.5 M) and had a resistance of 0.5–0.8 MΩ. During current recording and compound testing the cell membrane was constantly clamped to − 80 mV. Current signals were recorded and stored on a PC-type computer using the ROBOOCYTE software.

#### Solutions

During the microinjection and up to the electrophysiological experiment the oocytes were kept in a solution containing 88 mM NaCl, 1 mM KCl, 2.5 mM NaHCO_3_, 10 mM HEPES (AppliChem GmbH, Darmstadt, Germany), 0.82 MgSO_4_, 0.33 mM Ca(NO_3_)_2_, 0.33 mM CaCl_2_, 100 U/mL penicillin (GIBCO, ThermoFisher Scientific) and 100 µg/mL streptomycine (GIBCO, ThermoFisher Scientific). The pH was adjusted to 7.5. During the experiment the voltage-clamped oocyte was constantly superfused by a solution containing 90 mM NaCl, 1 mM KCl, 5 mM HEPES (AppliChem), 1 mM MgCl_2_ and 1 mM CaCl_2_ (pH 7.4). GABA and test compounds were added to this solution. GABA concentrations were chosen according to previously determined concentration–response relations for the different human GABA_A_ receptor subunit combinations. Concentrations evoking approximately 10% of a maximal response were selected, i.e., 8 µM for α1β2γ2, 6 µM for α2β3γ2, 10 µM for α3β3γ2 and 5 µM for α5β3γ2. Solutions containing GABA or test compounds were freshly prepared at least every second day. Stock solutions of basmisanil, flumazenil and β-CCM were prepared in DMSO at 10 mM concentration. Compounds were further diluted in DMSO and finally in extracellular saline to reach a DMSO concentration of 0.1%. The same amount of DMSO was added to the control solutions without test compounds.

#### Compound application

During the experiment the solutions were fed into the well containing the voltage-clamped oocyte via Teflon tubings connected by electromagnetic valves which allowed rapid switching between different solutions (controlled by the ROBOCYTE software). Approximately 1–2 min after impaling an oocyte and switching to voltage-clamp mode GABA was applied for 90 s to record a control response. Thereafter the oocyte was washed with saline for 160 s followed by another 90 s GABA application. Then 30 s after the onset of the second GABA application the system switched to a solution containing GABA plus the test compound. Each oocyte was used for testing one concentration of a single test compound (for α5β3γ2: 1 nM: n = 8, 10 nM: n = 8, 100 nM: n = 7, 1000 nM: n = 7).

Experiments for testing the antagonism between basmisanil and flumazenil followed the same general schedule. In this case each GABA application lasted 110 s and the rest period between the two GABA applications was 190 s long. As before, basmisanil was added to the GABA-containing solution 30 s after the onset of the second GABA response and 20 s later the system switched to a solution containing GABA plus basmisanil plus flumazenil (basmisanil + flumazenil 30 nM: n = 6, basmisanil + flumazenil 1000 nM: n = 10).

On each 96-well plate 3–6 oocytes were used for testing a known NAM at the BZD binding site, β-CCM (FG7106), as a positive control. This compound does not discriminate between the different GABA_A_ receptor subtypes and thus could be used for all subunit combinations to see if the oocytes were normally responding. Maximally effective concentrations of β-CCM were used for each subunit combination, i.e., 100 nM β-CCM for α1β2γ2, 300 nM for α2β3γ2 or α3β3γ2 and 1 µM for α5β3γ2. The inhibitory effects of β-CCM ranged between 42 and 55% (mean value per plate; n = 5–7).

#### Function of cloned GABA_A_ receptors

To determine concentration–response relationships, we calculated for each oocyte the ratio of the GABA-induced currents recorded in the presence and absence of the test compound to estimate modulation of the GABA response induced by the compound. For obtaining concentration–response curves, the data was fitted by Eq. (1) with A = 0.

### In vivo studies

Basmisanil was formulated by the Roche Galenic Laboratories as a micro-suspension in a vehicle containing 1.25% HPMC (hydroxypropyl methylcellulose) and 0.1% DOSS (dioctyl sodium sulfosuccinate). Concentrations were chosen to achieve the desired doses by administering 5 mL/kg to rats and 2.5 mL/kg to NHPs via oral gavage. Pre-treatment times were selected for each species/test to correspond with maximum plasma concentrations of basmisanil, based on previously conducted pharmacokinetic experiments (for examples see Table 1, Supplementary Fig. S1, Table S3).

**Table 1:** Summary of mean pharmacokinetic parameters of basmisanil following single oral dosing across species.

Species (n/sex)	Dose (mg/kg)^a^	C_max_^b^ (ng/mL)	T_max_ (h) (range)	AUC_0-last_^b^ (ng*h/mL)	F (%)
Wistar rat (n = 2 male)	9	400	0.5	1100	41
C57/Bl6 mouse (n = 2 male)	4	520	0.3	455	25
Beagle dog (n = 3 male)^c^	4	788 ± 275	1.5 (0.8–5)	10,600 ± 2040	61
Cynomolgus macaque (n = 3 male)	10	3650 ± 110	1.5 (1.5–3)	27,500 ± 8800	48
Human (n = 6 male), SAD study BP25129	45 mg	767 ± 15.4	3.5 (2.0–4)	6040 ± 38.7	~ 75
	160 mg	1860 ± 14.3	4.0 (2.5–6)	20,300 ± 38.3	
	330 mg	2120 ± 24.0	4.0 (2.5–8)	30,500 ± 20.8	

*AUC* area under the concentration–time curve, *C*_*ma*x _maximum plasma concentration, *SAD* single ascending dose, *F* bioavailability (%), *T*_*max*_ median time to reach maximum concentration.

^a^Dose is expressed as mg/kg, except for humans where dose is expressed as mg.

^b^For dog and cynomolgus macaque: ± standard deviation; for human: ± percent coefficient of variation.

^c^Basmisanil administered as capsule in dog, as microsuspension in rat, mouse and cynomolgus macaque, and as tablet formulation in human.

### Animals

Experiments performed at F. Hoffmann-La Roche (Basel) complied with the Swiss Federal and Cantonal laws on animal research and AAALAC regulations and received prior approval by the Cantonal Veterinary Office. The object retrieval task in NHPs was approved by the Roche Palo Alto Institutional Animal Care and Use Committee and were in accordance with the NIH guidelines. All in vivo experiments were carried out in compliance with the ARRIVE guidelines^61^.

Rats were group housed in separate holding rooms at a controlled temperature (20–23 °C), humidity (55–65%) and with a 12 h light/dark cycle. They were allowed ad libitum access to food and water. The animals used in the experiments were Wistar rats (PTZ experiments: 200–220 g, male and female; Charles River, France), Sprague–Dawley rats (receptor occupancy: 180 g, female; stimulus rat for social approach avoidance test: 450–500 g, male; Iffa Credo, France), Lister Hooded rats (Morris water maze: 220–250 g, male; Harlan, Netherlands), F-344 Fischer rats (social approach avoidance test: 170–180 g, male; Charles River, Germany).

Male cynomolgus macaques (Macaca fascicularis; 7–10 kg; Charles River, Houston TX) were fed their full daily regimen of food (Purina High Protein Monkey Chow #5045) following testing and had access to water ad libitum. The animals were kept on a 12 h light/dark cycle and were given fresh fruits and vegetables daily in addition to their chow.

### Receptor occupancy in rats

To estimate in vivo GABA_A_-α5 receptor occupancy in rats, [^3^H]-Ro 15-4513 was employed as a radioligand using a similar experimental approach as described previously^14^. Female Sprague–Dawley rats were pretreated with vehicle or basmisanil at doses of 3, 10, 30 or 100 mg/kg p.o. (n = 4 per dose group, n = 2 for the 100 mg/kg group) and 45 min later received intravenously 0.1 mCi/kg of [^3^H]-Ro 15-4513 (equivalent to a dose of 0.6 µg/kg).

For determination of non-specific binding of [^3^H]-Ro 15-4513 one group of animals (n = 4) received the established GABA_A_-α5 blocker L-655,708 (10 mg/kg) i.p. 30 min prior to radioligand injection (see^62^). L-655,708 has 50 to 100-fold selectivity for GABA_A_-α5 over other GABA_A_-receptor subtypes. However, at a dose of 10 mg/kg this compound will—to some extent—also block non-α5 receptor subtypes. Rats were sacrificed by decapitation 15 min after administration of the radioligand, i.e., 60 min after administration of basmisanil. Brains and blood were collected from each animal. Brains were divided in two halves along their sagittal axis and frozen in dry ice. Blood plasma was used for the determination of the plasma concentrations of basmisanil. Half of the brain was used for autoradiography, as described earlier^14^. To obtain the specific binding (S), the radioactivity in the hippocampus of L-655,708-treated animals was subtracted from the radioactivity in the hippocampus of vehicle- and basmisanil-treated animals. The percentage of receptor occupancy (y) was calculated from:

$$ {\text{y }} = { 1}00 \, \left( {{1 }{-}{\text{ S}}_{{{\text{basmisanil}}}} } \right)/{\text{S}}_{{{\text{vehicle}}}} . $$

Quantitative autoradiography signals in the hippocampus were plotted against individual plasma concentrations of basmisanil. The curve was fitted to the experimental results using the Hill equation (Eq. (1) with Hill coefficient D = 1).

### Diazepam-induced spatial learning impairment in the Morris water maze

The current study used a modified version of the standard acquisition protocol, in which pre-trained rats had to learn to locate a new platform position during six trials on the test day, as described previously^14^. Subjects were semi-randomly divided into treatment groups based on their performance on the last training day. A computer tracking system (HVS Image Ltd, UK) was used to analyse each rat’s swim path on-line.

Basmisanil (3 or 10 mg/kg p.o.) was administered to male Lister Hooded rats 30 min before testing (n = 10/dose group). Diazepam was prepared in 0.3% Tween 80 v/v 0.9% saline and was administered i.p. 30 min prior to testing. Latency, pathlength and swim speed (Supplementary Fig. S2) were analysed with a one-factor ANOVA. For the probe trial, to determine whether each treatment group had learned the spatial position of the platform, within-subjects ANOVA was used to compare percent time spent in the platform quadrant with the left, right and opposite quadrants. All significant cases were followed by a post hoc Newman–Keuls test. In addition, a comparison between vehicle treated and diazepam-treated rats on the percent time spent in the platform quadrant was analysed using an unpaired t-test.

For estimating plasma levels at the time of testing, a separate cohort of rats was treated with basmisanil as described previously and were euthanized by decapitation either at 30 min (corresponding to start of testing) or 100 min (corresponding to end of testing) post-administration of the compound (n = 4 per dose and time point). Plasma samples were collected and frozen at − 80 °C for later analysis.

### Object retrieval task in cynomolgus macaques

Twelve cynomolgus macaques were presented with a clear acrylic box with one open side baited with a food treat, as described previously^14,38^. The test is easy when the open side of the box is directed towards the subject and difficult when the open sides face other directions. In difficult trials, subjects obtained the treat on the first attempt approximately 50% of the time. Basmisanil (1, 3, 10, 30 mg/kg) was administered 2 h prior to testing (n = 12/dose group; within-subjects design). Compound doses were administered in a pseudorandom order. Administration of dose and behavioral measurements were completed blind. Data were analysed using a one-way repeated measures ANOVA followed, in significant cases, by a post hoc Dunnett’s test. Plasma samples were collected from the same three subjects at each dose group at 3 h post administration.

### Pentylenetetrazole test

In this assay, basmisanil was administered in combination with a threshold dose of the convulsant PTZ which was identified in previous experiments in our laboratory (55 mg/kg, males; 70 mg/kg, females). PTZ was prepared in 0.9% saline and was administered s.c. in a volume of 1 mL/kg. The positive control FG7142, a non-selective GABA_A_ receptor NAM, was prepared in 0.3% Tween 80 v/v saline and was administered i.p. in a volume of 1 mL/kg. Immediately following PTZ administration, rats were placed into individual observation chambers (24 × 39 × 18 cm; Macrolon Type III cages) with sawdust coated floors and were observed for a 30 min period by an observer blind to the treatment (n = 8 rats per dose group; randomised). During this period the number of rats showing tonic convulsions was noted.

Basmisanil (3, 10, 30, 100 mg/kg) was administered to male rats 1 h prior to PTZ administration. In a separate experiment, basmisanil was administered to female rats 2 h (30, 100 mg/kg) or 3 h (300, 600 mg/kg) prior to PTZ administration. FG7142 was administered at 10 mg/kg 30 min prior to PTZ. The percent of rats in each dose group showing tonic convulsions was compared to the control group by the Pearson Chi-Square test.

Following tonic convulsions, rats were immediately euthanized by decapitation. All other rats were euthanized by decapitation at the end of the 30 min observation period. Plasma samples were collected and frozen at − 80 °C for analysis of drug content.

### Social approach avoidance

Experiments on social approach avoidance were performed as described previously by^41^ and^14^. A tracking system (Ethovision, Noldus, The Netherlands) was used to analyse each rat’s pathway.

Basmisanil was administered to male Fischer rats 1 h prior to testing at 3, 10, 30 and 100 mg/kg p.o. (n = 10 per treatment group; randomised). The positive control, Ro 19-4603, was suspended in 0.3% Tween 80 v/v 0.9% saline and administered at 3 mg/kg p.o. 30 min prior to testing. All basmisanil dose groups were compared to the corresponding control group using a one-way ANOVA followed, in significant cases, by Dunnett’s post hoc test. The effects of the reference compound, Ro 19-4603, was compared with vehicle using an unpaired t-test.

At the end of the test session rats were immediately euthanized by decapitation. Plasma samples were collected and frozen at − 80 °C for analysis of drug content.

### Bioanalysis

During this drug development project, we have analyzed the pharmacokinetics profile of basmisanil in rodents at different dose-ranges and formulations, as well as measuring plasma exposures from different rat and mouse strains following behavior testing. Based on the sum of this information, it was considered that n = 3–4 plasma samples per dose group is sufficient to estimate receptor occupancy in the behavior tests described in this manuscript. Free plasma concentrations were used to estimate receptor occupancy. Plasma protein binding of basmisanil was moderate in all species tested and was in the range of 86% (free fraction: 14%) in rats and 94.5% (free fraction: 5.5%) in primates, including humans.

Basmisanil plasma concentrations were analyzed from a column-switching HPLC method and turbo ion spray tandem quadrupole mass spectrometry (LC–MS/MS) detection with lower quantification limits of 1.0 ng/mL. The calibration range was from 1.0 to 5000 ng/mL. After protein precipitation with ethanol, the sample solution was injected onto a Gemini C18 trapping column (2 × 10 mm; Phenomenex, Torrance, CA, USA) and polar endogenous compounds and salts were rinsed off. Analytical separation was performed on an Atlantis T3 column (2.1 × 50 mm) (Waters, Milford, MA, USA), using methanol/water containing 0.1% formic acid as mobile phase. A drug analogue was used as an internal standard.

### Healthy volunteer studies

The healthy volunteer PET and EEG studies were conducted in accordance with the International Conference on Harmonisation (ICH) guideline for Good Clinical Practice (GCP) and the principles of the Declaration of Helsinki. All participants provided written informed consent prior to the commencement of each study.

Basmisanil was provided as an uncoated, immediate-release tablet formulation exhibiting four different dose strengths (0.5, 5, 40, and 250 mg) as described by^63^. Dose-ranges for the PET and EEG studies were selected based on the demonstration of safety and tolerability in a previous placebo-controlled, single ascending dose study completed in healthy male subjects (clinicaltrials.gov identifier: NCT01684891).

### PET receptor occupancy study

The PET study was undertaken as part of Protocol BP25611 and after four protocol amendments was described as: “A single-center, single blind molecular and functional imaging study to assess GABA_A_-α5 receptor expression, occupancy and functional connectivity in the brains of individuals with Down syndrome and healthy controls following a single oral administration of RO5186582 or placebo”. Full details of the protocol can be found in the Supplementary Information. The study was registered on August 17, 2012 with clinicaltrials.gov identifier NCT01667367. The study was approved by the North London Research Ethics Committee (REC) 3 (UK), reference 10/H0709/90, and permission to administer the radiotracer [^11^C]-Ro 15-4513 was obtained from the Administration of Radioactive Substances Advisory Committee of the UK (Ref: 630/3764/28825). The primary objectives of this study was to measure basmisanil occupancy at the GABA_A_-α5 subunit-containing receptors in the human brain after oral dosing, and to relate those measures to plasma concentrations of basmisanil to investigate the relationship between the plasma concentration and its target occupancy.

### Subjects

Subjects were screened and recruited at Hammersmith Medicine Research, and imaging assessments were conducted at the Imanova Centre for Imaging Sciences, London, UK. Nine healthy volunteers (7 males and 2 females, aged 21–39 years; Supplementary Table S6) were enrolled into the study. Eligible participants were healthy male and female subjects of any ethnic origin, aged between 18 and 40 years inclusive, and with a body mass index between 18 and 40 kg/m^2^ inclusive. Subjects with a medical history which could have predisposed them to convulsions (e.g., history of epilepsy) were specifically excluded as a precaution against the theoretical pro-convulsive risk from basmisanil. The number of subjects and the number of scans per subject were limited for ethical reasons, in order to keep the radiation exposure to volunteers as low as reasonably possible, whilst still achieving the study objective. The first subject entered the study on August 9, 2012 and the last subject entered the study on October 11, 2013. The study also included 4 subjects with Down syndrome (data not shown).

### Experimental procedure

Each subject received a baseline [^11^C]-Ro 15-4513 PET scan (PET scan 1). After the baseline scan, each subject received a single oral dose of basmisanil, followed by a further PET scan (PET scan 2). PET scan 2 took place approximately 3–5 h post dose (in order to coincide with the anticipated time of maximum concentration of basmisanil). Subjects returned for a follow-up visit approximately 7 to 10 days after their last dose of study medication.

A range of doses of basmisanil were evaluated: 20 mg (n = 2); 40 mg (n = 2); 80 mg (n = 1); 160 mg (n = 2); 1000 mg (n = 2). The study was single-blind, i.e., subjects were blinded to dose. Blood sampling was performed at four different timepoints starting 30 min before the post dose PET scan. Quantification of plasma basmisanil was performed by liquid chromatography-tandem mass spectrometry, with the lower limit of quantification at 1.0 ng/mL.

#### Radiochemistry

[^11^C]-Ro 15-4513 was prepared as described previously^64^.

#### Image acquisition

A structural T1-weighted magnetic resonance brain scan was acquired on a 3 T MRI scanner (Siemens Tim Trio 3 T; Siemens AG Medical Solutions, Erlangen, Germany). Data were acquired in the sagittal plane, using a 3D magnetization prepared rapid gradient echo (MP-RAGE) sequence with the following parameters: repetition time = 2300 ms, echo delay time = 2.98 ms, flip angle = 9°, isotropic voxels = 1.0 mm × 1.0 mm × 1.0 mm, 160 slices, total scanning time = 5 min, 03 s. MRI scans were inspected by a neuroradiologist to exclude subjects with any clinically relevant brain abnormalities.

Dynamic PET scans were acquired on a Siemens Biograph 6 PET/computed tomography scanner with TruePoint gantry (Siemens AG Medical Solutions, Erlangen, Germany). Subjects were positioned in the tomograph after insertion of a venous cannula in an antecubital vein, a head-fixation device was used to minimize movement during data acquisition. A low-dose computed tomography scan was performed before each injection of the radioligand for attenuation and scatter correction. Dynamic emission data were collected continuously for 90 min (frame durations: 8 × 15 s, 3 × 60 s, 5 × 120 s, 5 × 300 s, 5 × 600 s) after intravenous bolus injection of [^11^C]-Ro 15-4513 (range 264–314 MBq). Image data were reconstructed using Fourier rebinning and a 2D filtered discrete inverse Fourier transform algorithm with 5 mm isotropic Gaussian filter on a 128 × 128 matrix with 2.6 zoom giving 2 mm isotropic voxels. Corrections were applied for attenuation, randoms and scatter.

### Image data analysis

#### Image processing

Image data were analyzed using the MIAKAT software package (version 3.3.1). MIAKAT is implemented using MATLAB (version R2008b; The Mathworks Inc., Natick, MA, USA), and makes use of FSL (version 4.1.9; FMRIB, Oxford, UK^65,66^; functions for brain extraction and SPM5 (Wellcome Trust Centre for Neuroimaging, http://www.fil.ion.ucl.ac.uk/spm) for image segmentation and registration.

Each subject’s structural MRI image underwent brain extraction, grey matter segmentation and co-registration to a standard reference space (MNI152^67^. The MNI152 template brain image and associated atlas (CIC atlas^68^ was nonlinearly warped to the subject’s MR image to enable automated definition of regions of interest (ROIs).

The following ROIs were considered: occipital lobe, parietal lobe, temporal lobe, amygdala, hippocampus, parahippocampal gyrus, insular cortex, anterior cingulate, dorsolateral frontal cortex, medial frontal cortex, orbitofrontal cortex, globus pallidus, caudate, accumbens, putamen, midbrain, medulla, pons and ventral cerebellum. ROIs were selected to provide broad coverage of the brain, and encompass a range of levels of GABA_A_-α5 expression^69^ and [^11^C]-Ro 15-4513 signal (based on previous literature [e.g.^70^] and baseline data from this study).

Dynamic PET images were registered to each subject’s MRI scan and corrected for motion using a frame-to-frame registration process with a normalized mutual information cost function. ROIs defined on the MRI images were applied to the dynamic PET data to derive regional time-activity curves (TACs), with activity concentrations expressed as standardized uptake values (SUV), given by (Eq. (2)):

2 $$ {\text{SUV }} = {\text{ Act}}/\left( {{\text{IA}}/{\text{BW}}} \right), $$

where Act is the measured radioactivity concentration (kBq/ml), IA is the injected radioactivity (MBq) and BW is the subject’s bodyweight (kg).

#### Kinetic modelling

Tissue time activity curves were analyzed using the simplified reference tissue model (SRTM^71^), with a basis pursuit implementation of the SRTM^72^, to directly estimate the binding potential (BP_ND_) in all target regions. Though the pons has the lowest [^11^C]-Ro 15-4513 signal, it is thought to be poorly representative of target regions in terms of nonspecific binding. Therefore, the time-activity curve derived from the cerebellum was used as an input for SRTM analysis. Methods of quantification of [^11^C]-Ro 15-4513 have been previously reported^42^ test-rested variability for [^11^C]-Ro 15-4513 has also been reported^73^.

Occupancy in scan 2 was estimated as a fractional reduction in the relevant baseline BP_ND_ (see Eq. (3)).

3 $$ {\text{Occ }} = \, \left( {{\text{BP}}_{{{\text{ND}}}}^{{{\text{Baseline}}}} {-}{\text{ BP}}_{{{\text{ND}}}}^{{{\text{Basmisanil}}}} } \right)/{\text{BP}}_{{{\text{ND}}}}^{{{\text{Baseline}}}} . $$

#### Pharmacokinetic-target occupancy analysis

The pharmacokinetic/pharmacodynamic population for GABA_A_-α5 receptor occupancy consisted of data from 9 subjects. A simple Emax model was found to describe the data adequately. This model was fitted to the whole data set using GraphPad Prism, version 5.03 (GraphPad Software, San Diego CA) to obtain estimates of EC_50_: E = E_max_ × [basmisanil]/(EC_50_ + [basmisanil]).

### Healthy volunteer EEG

EEG was assessed during a drug-drug interaction (DDI) study of basmisanil with the non-selective GABA_A_ PAM, midazolam, under Protocol WP29393: “A non-randomized, open-label, five treatment, fixed sequence crossover study to investigate the effect of RO5186582 treatment on CYP3A4 activity in healthy volunteers”. Full details of the protocol can be found in the Supplementary Information. The study was registered on October 2, 2014 with clinicaltrials.gov identifier NCT02254759. The DDI study was approved by the National Research Ethics Service (NRES) Committee South Central—Berkshire B. EEG was included in the study as one of the pharmacodynamic outcome measures.

### Subjects

Twelve healthy volunteers (9 males and 3 females, aged 25–57 years; Supplementary Table S7) were screened and recruited at Covance Clinical Research Unit, Ltd, Leeds, UK. Eligible participants were healthy male and female subjects of any ethnic origin, aged between 18 and 60 years inclusive, and with a body mass index between 18 and 32 kg/m^2^ inclusive. Subjects with a medical history which could have predisposed them to convulsions (e.g., history of epilepsy) were specifically excluded as a precaution against the theoretical pro-convulsive risk from basmisanil. The study started on September 29, 2014 (first informed consent) and ended on November 24, 2014 (final post-study observation).

### Experimental procedure

#### Participants and experimental task

Resting state EEG (4 min eyes open, 4 min eyes closed) was recorded at 4 days 1 h and 4 h after dosing at morning (similar time at baseline day). Assessments days: baseline, midazolam (5 mg, oral administration), 14 days of basmisanil treatment (240 mg twice daily, oral administration, recording following morning dose at day 14), and after 16 days of basmisanil treatment (240 mg twice daily, oral administration, recording following morning dose at day 16) plus midazolam (5 mg, oral administration). The EEG recording from day 16 is not reported here. The EEG technician kept the subject alert (vigilance controlled resting state condition). As soon as drowsiness patterns appeared (slow rolling eye movements, attenuated alpha rhythm replaced by low amplitude), the subject was aroused by auditory stimuli (tapping or clicking).

#### Blood sampling

Blood samples for basmisanil were collected on Day 18 of the study, at scheduled times up to 10 h after the administration of midazolam and up to 12 h after the administration of basmisanil. Concentration data collected at the time corresponding to the maximum plasma concentration for basmisanil (4 h post-dose) were used for the purpose of the pharmacokinetic/pharmacodynamic analysis.

#### EEG recording

EEG was recorded from 19 Ag/AgCl electrodes arranged according to the 10/20 system and mounted in an elastic cap. EOG was recorded simultaneously from five electrodes. Data were acquired at sampling rate of 256 Hz (alpha trace digitalEEG EPV-32, Neuro Medical, Arnheim, Netherlands; high-pass: 0.16 Hz; low-pass filter: 70 Hz; line-noise notch filter at 50 Hz; recording reference POz). Impedances were kept below 20 kΩ.

#### Preprocessing

All pre-processing was performed blinded to the treatment. Eyes open and eyes closed resting state data sections were extracted, high-pass filtered (0.5 Hz, FIR filter, n = 2 × sampling rate), and line-noise notch-filtered (Butterworth filter 49–51 Hz, order 4). Data sections confounded by large technical artifacts (e.g. large-amplitude transients) or prominent physiological artifacts (e.g. strong muscle activity) where identified by visual inspection and excluded from further processing. Then independent component analysis was applied to all of each subject’s recordings from a day to remove remaining artifacts (artifactual ICs rejected: muscle, ECG, eye movements and blinks, technical artifacts, number of rejected ICs: 4.9 ± 1.94, mean ± SD, range 1–9). Finally, the data was re-referenced to average reference. Pre-processing resulted in 221 ± 26.7 s (mean ± SD, range 114–240 s) of data per subject and condition for analysis. Overall the data quality was suitable for the planned analyses judged by fraction rejected as artifacts and qualitative impression of EEG traces.

#### Individual alpha peak frequency

Individual alpha peak frequency was defined as the maximum in the frequency range of 7–12 Hz of the power spectrum extracted from eyes closed resting state data at baseline (FFT applied to artifact free data in a 1 s sliding window, 3/4 overlap, zero-padded to 25 s). The existence of an alpha peak was confirmed in all 12 subjects visually. As would be expected, alpha peak frequencies (9.3 ± 0.87 Hz, mean ± SD, range 8.2–0.7 Hz) varied substantially across subjects.

#### Power spectral estimates

Power spectral estimates were derived for logarithmically scaled frequencies with a logarithmic frequency smoothing using Morlet Wavelets with a spectral smoothing of 1/2 octave (f/σf = 5.83). Frequencies were spaced logarithmically according to the exponentiation of the base 2 with exponents ranging from 1 (2 Hz) to 6 (64 Hz) in steps of 1/8. Spectral estimates were derived in successive 3/4-overlapping temporal windows. This frequency transform accounts for the logarithmic nature of electrophysiological data^74^. The analysis accounted for intra-individual differences in alpha peak frequency by individually adapting frequencies (individual shifts of the center frequencies in log-space) to align all alpha peaks to the mean across subjects (9.3 Hz). This allowed us to pin-point possible effects relative to the alpha peak frequency. Power values where scaled to have units V^2^/log_2_(Hz). For analyses of “relative power” the power was normalized to 1 across the frequency range analyzed.

### Statistical analysis

Student's t-tests were used for single comparisons and to derive confidence intervals. To test for differences in spectral power across frequencies cluster randomization tests were employed^75^: Condition labels for the contrast of interest (e.g. baseline and drug) were randomized (n = 10,000), t-test were performed for each frequency (two-tailed), then clusters (contiguous frequency ranges with p-values below the threshold of 0.01) were identified, and the size of the largest cluster across the entire frequency space from each randomization was used to generate a Null-hypothesis distribution. Cluster-randomization statistics accounts for multiple-comparison across all frequencies as well as positive and negative changes (two-tailed) in a data-adaptive manner.

We derived individual estimates of peak frequencies of pharmacological effects using pseudo-values^76^ in order to (a) estimate the standard error of the mean of the peak frequency of effects and (b) to compare peak frequencies of effects induced by midazolam and basmisanil.

To investigate possible differences in the spatial topography of drug effects (reflective on the spatial localization of the underlying neuronal populations), we compared the average mutual correlation between all subjects’ topographies for basmisanil and the average mutual correlation between all subjects’ topographies induced by midazolam to the correlation of topographies between drugs. Significance was assessed by means of a randomization test.

Any values reported in the format < number >  ±  < number > are mean ± standard deviation for the given quantity.

All analyses were performed in Matlab with custom scripts and the Fieldtrip toolbox to generate topographic plots.

## Supplementary Information

Supplementary Information.
