# Supplementary material for: Basmisanil, a highly selective GABAA-α5 negative allosteric modulator: preclinical pharmacology and demonstration of functional target engagement in man
Source: Sci Rep. 2021 Apr 8;11:7700. doi: 10.1038/s41598-021-87307-7 (PMC8032764; doi:10.1038/s41598-021-87307-7)
Supplement: Supplementary file 1 — Supplementary Information. [file 41598_2021_87307_MOESM1_ESM.pdf]

Supplementary Information

Basmisanil, a Highly Selective GABA$_A$-α5 Receptor Negative Allosteric Modulator: Preclinical Pharmacology and Demonstration of Functional Target Engagement in Man

Joerg F Hipp[1], Frederic Knoflach[1], Robert Comley[1], Theresa M. Ballard[1], Michael Honer[1], Gerhard Trube[1], Rodolfo Gasser[2], Eric Prinssen[1], Tanya L. Wallace[5], Andreas Rothfuss[2], Henner Knust[3], Sian Lennon-Chrimes[4], Michael Derks[4], Lisa Squassante[4], Stephane Nave[1], Jana Nöldeke[1], Christoph Wandel[2], Andrew W Thomas[3], Maria-Clemencia Hernandez[1]

*Corresponding author: maria-clemencia.hernandez@roche.com

Table of contents

Page	Item	Content
3	**Figure S1**	Pharmacokinetic profile of basmisanil in cynomolgus macaque and Wistar rat.
4	**Figure S2**	Effect of diazepam alone and following pre-treatment with basmisanil during the six learning trials of acutely treated rats in the Morris water maze.
5	**Figure S3**	Basmisanil does not impair prepulse inhibition induced by different prepulse intensities (72, 76, 80, 84 dB).
6	**Figure S4**	Plasma exposures achieved with basmisanil in the rat PTZ test and in GLP toxicological studies in rats and dogs compared with plasma exposure required for full GABA$_A$ α5 receptor occupancy.
7	**Figure S5**	Electrophysiological signatures of midazolam and basmisanil.
9	**Table S1**	Restriction enzyme sites used to linearize the different GABA$_A$ receptor subunit cDNAs cloned into the pcDNA3.1 vectors.
10	**Table S2**	Selectivity profile of basmisanil.

13	**Table S3**	Summary of mean pharmacokinetic parameters of basmisanil following single intravenous dosing.
13	**Table S4**	Summary of the in vitro profile of $GABA_A$ α5 receptor NAMs
14	**Table S5**	Basmisanil doses, plasma concentrations and regional receptor occupancy values in human PET studies
15	**Table S6**	Summary of demographic characteristics of healthy volunteers in PET study
15	**Table S7**	Summary of demographic characteristics of healthy volunteers in EEG study
16	**Clinical information**	Safety EEG data from Phase I studies in healthy volunteers

a b

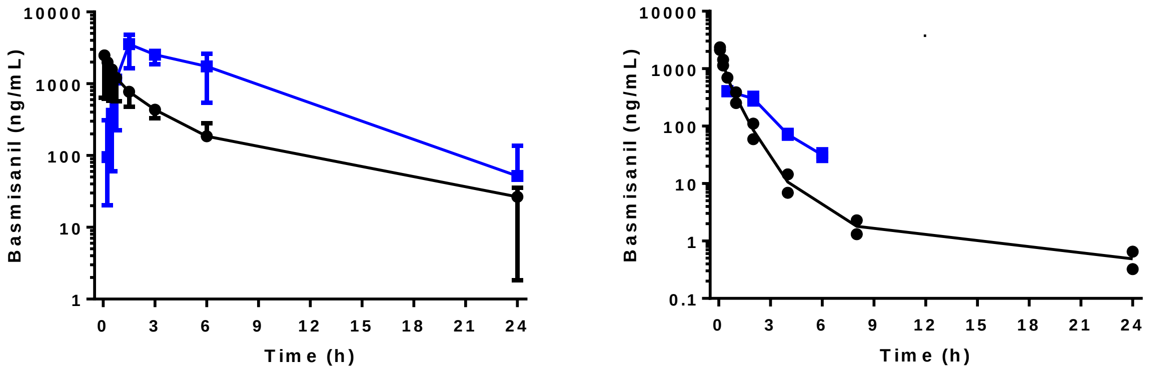

Figure S1. Pharmacokinetic profile of basmisanil in cynomolgus macaque and Wistar rat. (**a**) Pharmacokinetic profile of basmisanil in cynomolgus macaques after 1 mg/kg i.v. (blue line) and 10 mg/kg p.o administration (black line). (**b**) Pharmacokinetic profile of basmisanil in male Wistar rats after 4.3 mg/kg i.v. (blue line) and 9 mg/kg p.o. administration (black line). Data are mean ± S.D.; n = 2–3 per species and route.

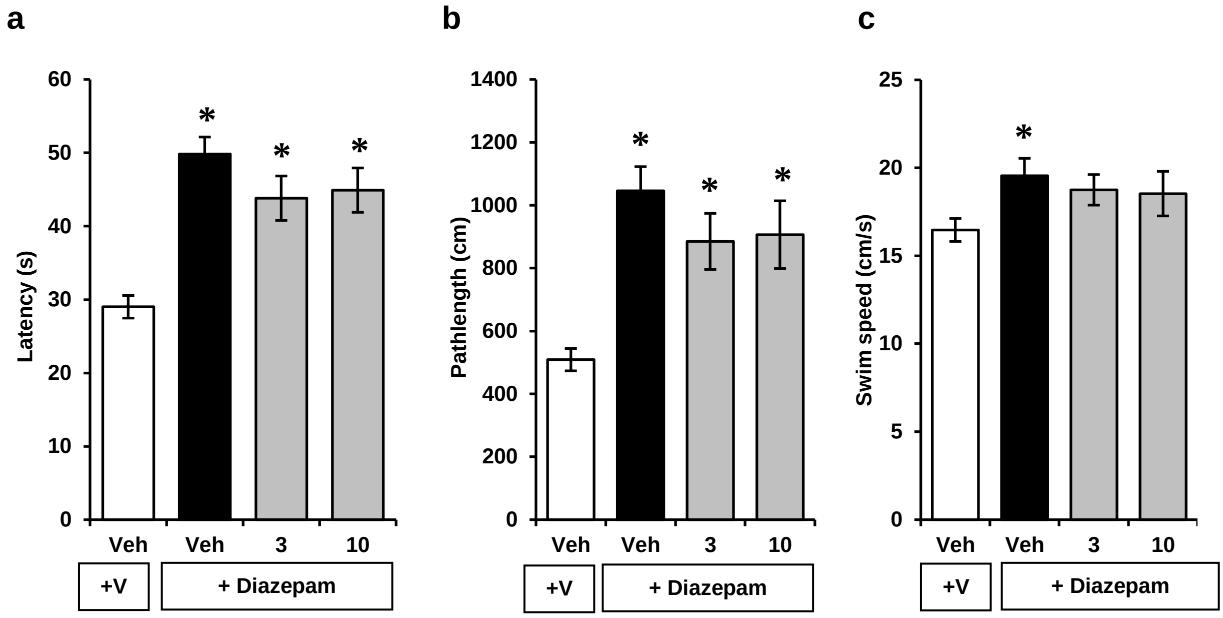

Figure S2. Effect of diazepam alone and following pre-treatment with basmisanil during the six learning trials of acutely treated rats in the Morris water maze.
(**a**) latency (s) to find platform position; (**b**) pathlength (cm) travelled to find platform position; (**c**) swim speed (cm/s). Abscissa labels: Veh or V = vehicle, Diazepam at 6 mg/kg i.p., 3 = 3 mg/kg p.o. basmisanil, 10 = 10 mg/kg p.o. basmisanil. Compounds were administered 30 min before the test. Bars indicate mean of 6 trials ± S.E.M. Statistics: * $p<0.05$ vs. vehicle-treated group; 10 male Lister hooded rats per dose group.

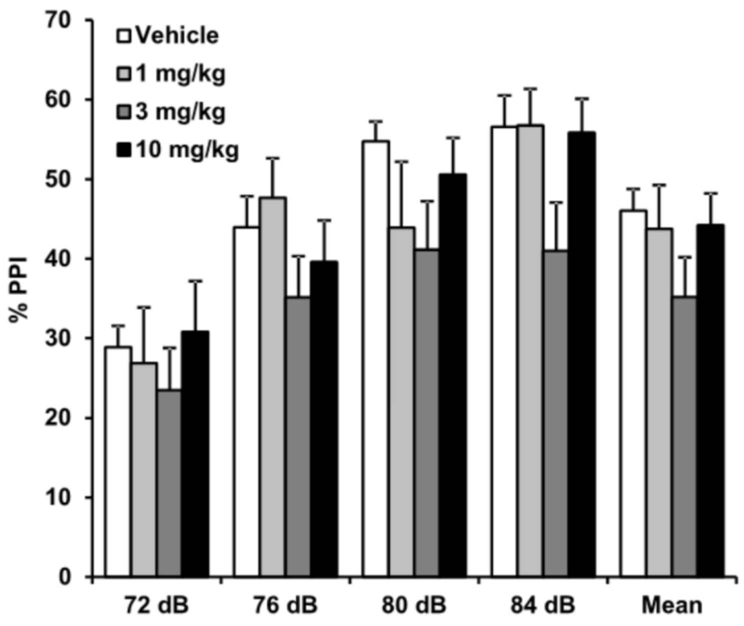

Figure S3. Basmisanil does not impair prepulse inhibition induced by different prepulse intensities (72, 76, 80, 84 dB) at total plasma concentrations of 56 ± 20, 169 ± 20, and 729 ± 255 ng/mL (mean ± SD).
Compound administered at 1, 3 and 10 mg/kg p.o. to male Sprague-Dawley rats 30 min prior to testing. Bars indicate mean percent prepulse inhibition (% PPI) ± SEM; 10 animals per dose group. Background noise: 68 dB. Startle pulse: 120 dB. For full methods refer to: Nordquist et al. (2008). Effects of aripiprazole/OPC-14597 on motor activity, pharmacological models of psychosis, and brain activity in rats. Neuropharmacology, 54,405-416.

Animals were euthanized at the end of testing and plasma samples were collected approximately 60 min post administration; n = 4 rats per group.

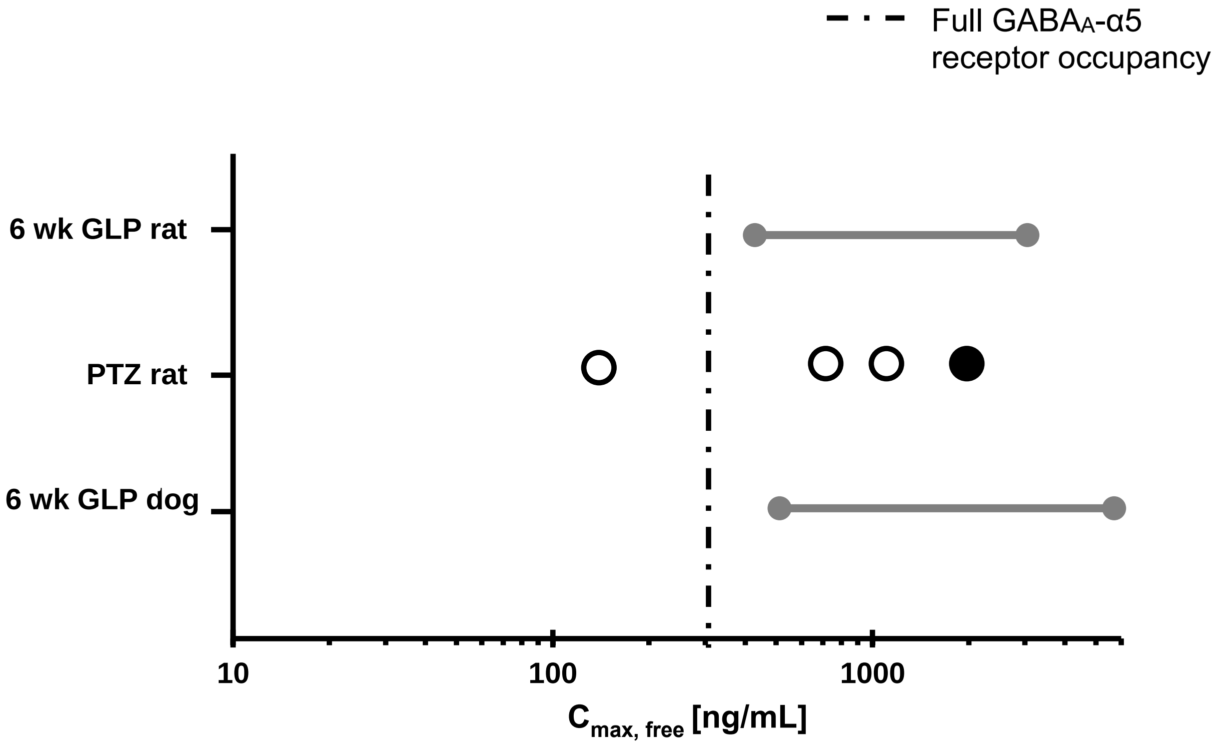

Figure S4. Plasma exposures achieved with basmisanil in the rat PTZ test and in GLP toxicological studies in rats and dogs compared with plasma exposure required for full $GABA_A$ α5 receptor occupancy (Rothuss et al., 2016; Wandel et al., 2015).
Grey lines: maximum free plasma concentration ($C_{max,free}$) range covered in GLP toxicology studies. Open circles: plasma exposures with no pro-convulsant activity in PTZ test. Closed circle: pro-convulsant activity in 5/8 rats in PTZ test.

References:

Rothfuss A, Wandel C, Thomas A, Knoflach F, Trube G, Husar E, Gasser R, Schmitt M, Noeldeke J, Hernandez M-C. Basmisanil, a negative allosteric modulator of the gamma-aminobutyric acid Aα5 receptor subtype, does not show convulsions at relevant exposures. The Toxicologist. 2016;151.

Wandel C, Thomas A, Hernandez M-C, Ballard-Yardy T, Knoflach F, Trube G, Rothfuss A, Husar E, Lennon-Chrimes S, Bentley D, Liogier d'Ardhuy X, Squassante L, Noeldeke J, Khwaja O. RG1662, a new negative allosteric modulator of the gamma-aminobutyric acid Aα5 receptor subtype, does not show convulsions at relevant doses. ECNP 2015; P.1.g.041.

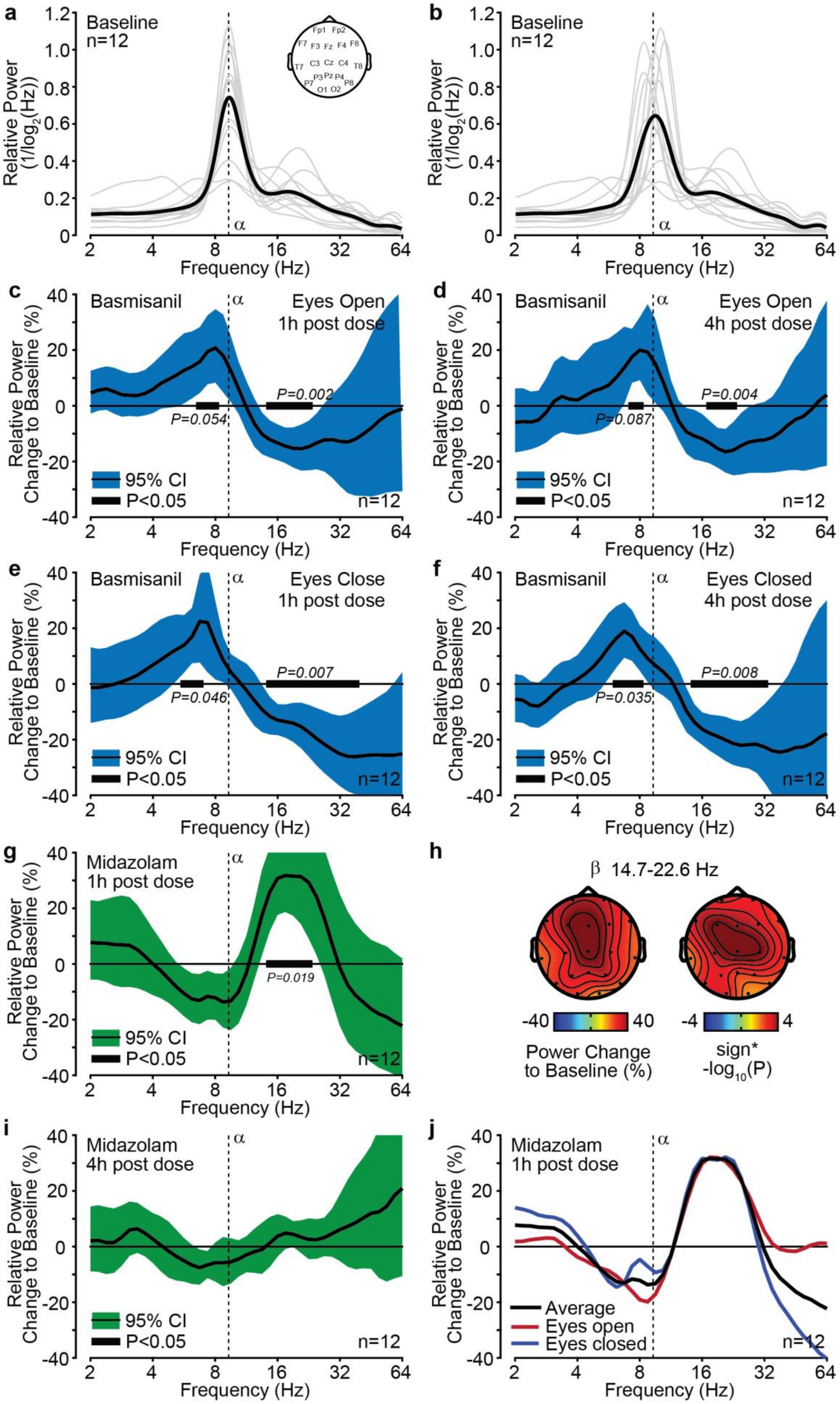

a
Baseline
n=12
Relative Power (1/log2(Hz))
Frequency (Hz)
Fp1 Fp2 F7 F3 Fz F4 F8 T7 C3 Cz C4 T8 P7 P3 Pz P4 P8 O1 O2
b
Baseline
n=12
c
Basmisanil
Eyes Open
1h post dose
P=0.002
P=0.054
95% CI
P<0.05
n=12
Relative Power Change to Baseline (%)
d
Basmisanil
Eyes Open
4h post dose
P=0.004
P=0.087
e
Basmisanil
Eyes Close
1h post dose
P=0.007
P=0.046
f
Basmisanil
Eyes Closed
4h post dose
P=0.008
P=0.035
g
Midazolam
1h post dose
P=0.019
h
β 14.7-22.6 Hz
Power Change to Baseline (%)
sign* -log10(P)
i
Midazolam
4h post dose
j
Midazolam
1h post dose
Average
Eyes open
Eyes closed

Figure S5. Electrophysiological signatures of midazolam and basmisanil.
(**a**) Baseline power spectrum, alpha peaks aligned (see methods). (**b**) Baseline power spectrum, alpha peaks not aligned. (**c-f**) Relative power change of EEG signals averaged across electrodes in response to basmisanil (eyes open or eyes closed conditions at 1 h or 4 h post morning dose after 14 days of 240 mg treatment twice daily). Black bars indicate significant effects for basmisanil (cluster randomization test). (**g**) Relative power change of EEG signals averaged across all electrodes in response to midazolam (1 h post a single 2 mg dose, average of eyes open and eyes closed). Black bar indicates significant effect (cluster randomization test) (**h**) Spatial distribution of power changes and p-values (derived from t-tests) for the significant cluster identified for Midazolam (14.7-22.6 Hz, black bar in **g**). Left: power changes, right: P-values. (**i**) Relative power change of EEG signals averaged across all electrodes in response to midazolam (4h post a single 2 mg dose, average of eyes open and eyes closed conditions). No significant effect (cluster randomization test). (**j**) Relative power change of EEG signals averaged across all electrodes in response to midazolam for eyes open and eyes closed conditions 1 h post a single 2 mg dose .
CI = confidence interval (uncorrected for multiple testing across frequencies).

Table S1. Restriction enzyme sites used to linearize the different $GABA_A$ receptor subunit cDNAs cloned into the pcDNA3.1 vectors

cDNA	Vector	Restriction site
GABRA1_HUMAN	pcDNA 3.1(-)	Hind III
GABRA2_HUMAN	pcDNA 3.1(+)	Xba I
GABRA3_HUMAN	pcDNA 3.1(-)	Hind III
GABRA5_HUMAN	pcDNA 3.1(-)	Hind III
GABRB2_HUMAN	pcDNA 3.1(+)	XbaI
GABRB3_HUMAN	pcDNA 3.1(-)	NotI
GABRG2_HUMAN	pcDNA 3.1(+)	XbaI
GABARAP_HUMAN	pcDNA 3.1(+)	NotI

Table S2. Selectivity profile of basmisanil.

Source: species (hum. = human) and tissue or cell line or recombinant (recomb.) receptors (cell line of expression in parentheses). **% Inhibition**: percentage inhibition of specific binding of the radioligand in presence of 10 μM basmisanil. Mean values of n = 2. All experiments were done by Eurofins Cerep SA, Celle l'Evescault, France. For more information see the website, www.neurofinsdiscoveryservices.com.

Receptor	Source	Ligand	% Inhibition
Non-peptide receptors			
Adenosine A_1	hum. recomb. (CHO)	[^{3}H]DPCPX	28
Adenosine A_{2A}	hum. recomb. (HEK-293)	[^{3}H]CGS 21680	9
Adenosine A_3	hum. recomb. (HEK-293)	[^{125}I]AB-MECA	6
Adrenergic α_1 (non-selective)	rat cerebral cortex	[^{3}H]prazosin	-1
Adrenergic α_2 (non-selective)	rat cerebral cortex	[^{3}H]RX 821002	-8
Adrenergic β_1	hum. recomb. (HEK-293)	[^{3}H](-)CGP 12177	6
Adrenergic β_2	hum. recomb. (CHO)	[^{3}H](-)CGP 12177	10
Benzodiazepine (central)	rat cerebral cortex	[^{3}H]flunitrazepam	76
Benzodiazepine (peripheral)	rat heart	[^{3}H]PK 11195	-10
Cannabinoid CB_1	hum. recomb. (CHO)	[^{3}H]CP 55940	16
Dopamine D_1	hum. recomb. (CHO)	[^{3}H]SCH 23390	0
Dopamine D_{2S}	hum. recomb. (HEK-293)	[^{3}H]spiperone	9
Dopamine D_3	hum. recomb. (CHO)	[^{3}H]spiperone	3
Dopamine $D_{4.4}$	hum. recomb. (CHO)	[^{3}H]spiperone	9
Dopamine D_5	hum. recomb. (GH4)	[^{3}H]SCH 23390	-11
GABA (non-selective)	rat cerebral cortex	[^{3}H]GABA	-1
Histamine H_1	hum. recomb. (HEK-293)	[^{3}H]pyrilamine	10
Histamine H_2	hum. recomb. (CHO)	[^{125}I]APT	8
Melatonin MT_1 (ML_{1A})	hum. recomb. (CHO)	[^{125}I]2-iodomelatonin	14
Muscarinic M_1	hum. recomb. (CHO)	[^{3}H]pirenzepine	6
Muscarinic M_2	hum. recomb. (CHO)	[^{3}H]AF-DX 384	13
Muscarinic M_3	hum. recomb. (CHO)	[^{3}H]4-DAMP	4
Muscarinic M_4	hum. recomb. (CHO)	[^{3}H]4-DAMP	-4
Muscarinic M_5	hum. recomb. (CHO)	[^{3}H]4-DAMP	-7
Prostanoid EP_4	hum. recomb. (CHO)	[^{3}H]PGE_2	3
Prostanoid TP (TXA_2/PGH_2)	hum. recomb. (HEK-293)	[^{3}H]SQ 29548	5
Prostanoid IP (PGI_2)	hum. recomb. (HEK-293)	[^{3}H]iloprost	-1
Purinergic P2X	rat urinary bladder	[^{3}H]α,β-MeATP	-7

Table S2 - continued			
**Receptor**	**Source**	**Ligand**	**% Inhibition**
Purinergic P2Y	rat cerebral cortex	[^{35}S]dATPαS	9
Serotonin 5-HT_{1A}	hum. recomb. (HEK-293)	[^{3}H]8-OH-DPAT	0
Serotonin 5-HT_{1B}	rat cerebral cortex	[^{125}I]CYP	0
Serotonin 5-HT_{2A}	hum. recomb. (HEK-293)	[^{3}H]ketanserin	-9
Serotonin 5-HT_{2B}	hum. recomb. (CHO)	[^{125}I](±)DOI	31
Serotonin 5-HT_{2C}	hum. recomb. (CHO)	[^{3}H]mesulergine	10
Serotonin 5-HT_{3}	hum. recomb. (CHO)	[^{3}H]BRL 43694	8
Serotonin 5-HT_{5a}	hum. recomb. (CHO)	[^{3}H]LSD	5
Serotonin 5-HT_{6}	hum. recomb. (CHO)	[^{3}H]LSD	7
Serotonin 5-HT_{7}	hum. recomb. (CHO)	[^{3}H]LSD	13
Sigma σ (non-selective)	rat cerebral cortex	[^{3}H]DTG	58
Peptide receptors			
Angiotensin AT_{1}	hum. recomb. (HEK-293)	[^{125}I][Sar^{1},Ile^{8}]-AT-II	2
Angiotensin AT_{2}	hum. recomb. (CHO)	[^{125}I]CGP 42112A	-3
Bombesin (non-selective)	rat cerebral cortex	[^{125}I][Tyr^{4}]bombesin	-24
Bradykinin B_{2}	hum. recomb. (CHO)	[^{3}H]bradykinin	14
Calcitonin gene related peptide (CGRP)	hum. recomb. (CHO)	[^{125}I]hCGRPα	-6
Cholecystokinin CCK_{1}	hum. recomb. (CHO)	[^{125}I]CCK-8s	10
Cholecystokinin CCK_{2}	hum. recomb. (CHO)	[^{125}I]CCK-8s	-21
Endothelin ET_{A}	hum. recomb. (CHO)	[^{125}I]endothelin-1	8
Endothelin ET_{B}	hum. recomb. (CHO)	[^{125}I]endothelin-1	-2
Galanin 1	hum. recomb. (HEK-293)	[^{125}I]galanin	0
Galanin 2	hum. recomb. (CHO)	[^{125}I]galanin	-1
Platelet derived growth factor (PDGF)	mouse, Balb/c 3T3 cells	[^{125}I]PDGF BB	-17
Chemokine CXCR2 (IL-8B)	hum. recomb. (HEK-293)	[^{125}I]IL-8	-3
Chemokine CCR1	hum. recomb. (HEK-293)	[^{125}I]MIP-1a	-2
Melanocortin MC_{4}	hum. recomb. (CHO)	[^{125}I]NDP-α-MSH	9
Neurokinin NK_{1}	hum., U-373MG cells	[^{125}I]BH-SP	37
Neurokinin NK_{2}	hum. recomb. (CHO)	[^{125}I]NKA	7
Neurokinin NK_{3}	hum. recomb. (CHO)	[^{3}H]SR 142801	-9
Neuropeptide Y_{1}	hum., SK-N-MC cells	[^{125}I]peptide YY	0
Neuropeptide Y_{2}	hum., KAN-TS cells	[^{125}I]peptide YY	7

Table S2 - continued			
**Receptor**	**Source**	**Ligand**	**% Inhibition**
Neurotensin NTS_1 (NT_1)	hum. recomb. (CHO)	[^{125}I]Tyr^3--neurotensin	-6
Opioid δ_2	hum. recomb. (CHO)	[^{3}H]DADLE	8
Opioid κ	hum. recomb. (CHO)	[^{3}H]U 69593	13
Opioid μ	hum. recomb. (HEK-293)	[^{3}H]DAMGO	3
Nociceptin (ORL1)	hum. recomb. (HEK-293)	[^{3}H]nociceptin	-4
Somatostatin (non-selective)	mouse, AtT-20 cells	[^{125}I]Tyr^{11}--somatostatin-14	18
Vasoactive intestinal peptide PAC_1 (PACAP)	hum. recomb. (CHO)	[^{125}I]$PACAP_{1-27}$	-16
Vasoactive intestinal peptide $VPAC_1$ (VIP_1)	hum. recomb. (CHO)	[^{125}I]VIP	0
Vasopressin V_{1A}	hum. recomb. (CHO)	[^{3}H]AVP	13
Ion channels			
Ca^{2+} channel, L-type (phenylalkylamine site)	rat cerebral cortex	[^{3}H](-)D 888	-3
K_v channel	rat cerebral cortex	[^{125}I]α−dendrotoxin	-1
SK_{Ca} channel	rat cerebral cortex	[^{125}I]apamin	3
Na^+ channel (site 2)	rat cerebral cortex	[^{3}H]batrachotoxinin	0
$GABA_A$ Cl^- channel (non- selective)	rat cerebral cortex	[^{35}S]TBPS	-6
NMDA (PCP)	rat cerebral cortex	[^{3}H]TCP	14
Amine transporters			
Norepinephrine transporter	hum. recomb. (CHO)	[^{3}H]nisoxetine	11
Dopamine transporter	hum. recomb. (CHO)	[^{3}H]BTCP	19
Serotonin transporter	hum. recomb. (CHO)	[^{3}H]imipramine	5
Nuclear receptor			
Glucocorticoid	hum., IM-9 cells, cytosol	[^{3}H]dexamethasone	-2

Table S3. Summary of mean pharmacokinetic parameters of basmisanil following single intravenous dosing.

Species, Strain (n)	Dose (mg/kg)	Total CL (mL/min/kg)	V_{ss} (L/kg)	$t_{1/2}$ (h)
Rat, Wistar (2 male)	4.3	54	2.9	1.2
Mouse, C57/Bl6 (2 male)	2.0	37	1.3	0.5
Dog, Beagle (n=3)	1.0	2.2 ± 0.2	1.7 ± 0.2	9.8 ± 0.8
NHP, Cynomolgus macaque (n=3)	1.0	3.0 ± 0.7	0.9 ± 0.6	4.5 ± 1.8

CL = clearance; V_{ss} = volume of distribution at steady-state; $t_{1/2}$ = terminal half-life.

Table S4. Summary of the in vitro profile of $GABA_A$-α5 NAMs

a. Radioligand binding

Compound	GABA$_A$ receptor subtype (Ki nM)					Reference
	α1β3γ2	α2β3γ2	α3β3γ2	α5β3γ2	α1/ α5 fold selectivity	
**Basmisanil**	1031	458	510	5.1	**202**	1)
RO4938581	174	185	80	4.6	38	2)
L-655,708	70	48	31	1.0	70	3)
**α5IA**	0.9	0.6	0.6	0.7	1	4)
**MRK-016**	0.8	0.9	0.8	1.4	1	5)
PWZ-029	920	>300	>300	30.0	31	6)
ONO-8290580	140	32	24	7.9	18	7)

b. Electrophysiological measurements

Compound	GABA$_A$ receptor subtype (% inhibition)					References
	α1β3γ2	α2β3γ2	α3β3γ2	α5β3γ2	Expression system	
**Basmisanil**	-5	-7	-1	-39.4	a)	1)
RO4938581	5.8	-9.9	-2.7	-43.3	b)	2)
L-655,708	-18	-23	-11	-17.0	c)	3)
**α5IA**	-18.0	13.0	-7.0	-40.0	c)	4)
**MRK-016**	-16.0	6.0	-9.0	-55.0	c)	5)
PWZ-029	14	5	18	-20.0	a)	6)
ONO-8290580	-3	5	-5	-44.4	b)	7)

References: 1) This manuscript 2) Ballard et al., 2008 3) Atack et al., 2006 4) Dawson et al., 2006 5) Atack et al. 2009 6) Savić et al., 2008 7) Kawaharada et al., 2018

Expression systems: a) Xenopus oocytes b) HEK293 cells c) Mouse L(tk-) cells

Table S5. Basmisanil doses, plasma concentrations and regional receptor occupancy values in healthy human volunteer PET studies

Subject ID	Dose (mg)	Time of dose (h prior to scan)	Basmisanil in plasma (ng/mL) at start of scan	Receptor occupancy (%)					
				Amyg dala	Hippo campus	Insular cortex	Anterior cingulate	Accumbens	Mean
**13040**	20	1.48	111	30.9	26.8	26.7	26.2	29.6	28.04
**13041**	20	2.20	398	34.7	35.8	31.7	31.7	31.8	33.14
**13010**	40	2.63	422	40	41.3	32	32.7	37.3	36.66
**11010**	40	3.75	592	53.5	48.4	44.6	44.8	52.1	48.68
**13050**	80	2.43	1000	63.5	63	57.8	55.8	64.2	60.86
**11020**	160	3.12	2280	91.4	87.2	75.2	75.6	86.6	83.2
**11021**	160	3.00	2550	85.4	85.9	77.2	76.4	87.0	82.38
**13030**	1000	4.75	2200	93.1	94.2	79.9	79.1	89.5	87.16
**13031**	1000	4.77	3350	97.4	94.9	82.2	82.1	93.7	90.06

Table S6. Summary of demographic characteristics of healthy volunteers in PET study

Age (year)	N = 9
Mean (SD)	25.3 (5.6)
Median	23.0
Min - Max	21 - 39
**Sex**	
Male	7
Female	2
**Race**	
Asian	2
Black or African American	1
White	6
**Weight (kg)**	
Mean (SD)	78.58 (8.75)
Median	77.80
Min - Max	60.2 - 91.4
**Height**	
Mean (SD)	173.67 (11.02)
Median	176.0
Min - Max	153.0 - 187.0
**Baseline Body Mass Index (kg/m^2)**	
Mean (SD)	26.47 (5.50)
Median	24.87
Min - Max	19.0 – 36.6

Table S7. Summary of demographic characteristics of healthy volunteers in EEG study

Age (year)	N = 12
Mean (SD)	42.6 (11.31)
Median	47.0
Min - Max	25 - 57
**Sex**	
Male	9
Female	3
**Race**	
White	12
Hispanic	0
Non-hispanic	12
**Weight (kg)**	
Mean (SD)	76.27 (10.96)
Median	75.40
Min - Max	52.4 – 97.6
**Height**	
Mean (SD)	175.5 (8.93)
Median	175.0
Min - Max	160.0 - 192.0
**Baseline Body Mass Index (kg/m^2)**	
Mean (SD)	24.71 (2.70)
Median	25.28
Min - Max	20.47 – 29.14

CLINICAL INFORMATION: Safety EEG data from Phase I studies in healthy volunteers

1) Study BP25129 Single-Ascending Dose (SAD) in Healthy Volunteers

Single doses of between 1.5 mg and 1250 mg were administered to groups of eight healthy male subjects (age range 18–44 years) per dose level (6 received active drug, two received placebo). All nine planned cohorts in the Part 1 SAD study were completed, and all subjects completed dosing as planned.

Food Effect (FE) Cohort: Eight subjects were enrolled, all received active drug as planned on two separate occasions, with food condition (Fed or Fasted) on each occasion determined in a randomized manner. All subjects enrolled in the FE portion of Part 1 completed the study.

EEG assessments were made in Cohorts 6, 8, 9 and FE Cohort only:

- Cohort 6: 160 mg (n dosed: 6)
- Cohort 8: 660 mg (n dosed: 6)
- Cohort 9: 1250 mg (n dosed: 6)
- Food effect Cohort: 660 mg (n dosed: 6)

A screening assessment on admission was carried out to exclude subjects with nonspecific EEG patterns and facilitate the interpretation of post dosing EEG recordings. The duration of the entire recording was approximately 15–20 minutes. In addition to recording in the resting awake state, this assessment included reaction to intermittent photic stimulation and hyperventilation. The two latter methods were designed to evoke abnormal EEG waveforms or rhythms typically associated with epileptogenesis resulting from an underlying abnormality.

On the day of dosing, an EEG in the resting, awake state was recorded starting about 1 hour prior to dosing and ending approximately 7 h after dosing. EEG results were recorded either as normal, normal variant or abnormal variant. If an abnormal variant measurement was recorded, then the Investigator could continue recordings as appropriate until a return to normal was reached.

Review of the EEG data was performed in an unblinded manner by external experts and Roche internal experts who were independent of the study project team.

Outcome:

Basmisanil was well tolerated. EEG assessments did not reveal any findings of clinical significance following single doses of 160 mg, 660 mg and 1250 mg with maximum plasma concentrations of 1860, 3710, and 4890 ng/mL.

2) Study WP25366 Multiple-Ascending Dose (MAD) in Healthy Volunteers

Of the 32 healthy male subjects who were randomized to treatment, 24 subjects were assigned to active treatment with basmisanil (6 subjects each received treatment with either basmisanil 80, 160, 370, or 1000 mg BID) and 8 subjects were randomized to placebo. All participants had several EEGs and several participants had sleep assessments with EEG that were also evaluated:

- Cohort 1: 80 mg (n dosed: 6)
- Cohort 2: 160 mg (n dosed: 6)
- Cohort 3: 370 mg (n dosed: 6)
- Cohort 4: 1000 mg (n dosed: 6)

EEG monitoring was implemented in this study starting at about 1 hour prior to dosing and then up to approximately 7 hours after dosing, on selected days during the dosing period. All sleep recordings were performed for approximately 8 h from 23.00 to 07.00. The objective of such monitoring was detection if any, of specific EEG patterns such as spikes, sharp waves, and spike-and-wave discharges that would be indicative of epileptiform changes or non-convulsive seizure.

The day and night EEG recordings were subject to expert safety review, with the objective of identifying any pro-convulsive signals. Following this safety review, EEG results were recorded either as normal, normal variant or abnormal variant.

Outcome:

EEG assessments revealed no findings of clinical concern, specifically any signs indicative of epileptogenic effects (e.g., generalized spike-wave discharges) in any subject in any dose group at any time points during the study. The review of the day and night EEG recordings did not reveal any clinically significant finding especially no abnormalities which could indicate that basmisanil has proconvulsive property. In this study basmisanil, at 80 mg BID, 160 mg BID, 370 mg BID and 1000 mg BID, was safe and had no impact on brain electrical activity. Maximum plasma concentrations at Day 13 were 2120, 4110, 6040, and 8520 ng/mL respectively.

<u>3) Study WP29402 Drug-Drug Interaction (DDI) Itraconazole in Healthy Volunteers</u>

This was an open-label, three treatment, fixed sequence crossover study to investigate the effect of CYP3A inhibition (itraconazole) on basmisanil exposure, safety and tolerability in healthy male and female volunteers (age range 18-60 years; n: 12). As systemic basmisanil exposure was predicted to be higher than exposures achieved in previous clinical studies, regular EEG examinations were included and reviewed by a neurologist. Multiple oral doses of basmisanil (120 and 240 mg BID, 10 days) were administered alone and co-administered with the CYP3A inhibitor, itraconazole (200 mg OD).

Baseline EEG assessments were performed prior to the study (resting EEG eyes open/closed, intermittent photic stimulation, hyperventilation). On treatment days (1, 3, 5, 8) during co-administration of basmisanil and itraconazole, continuous EEG monitoring for 6 hours and resting EEG eyes open/closed were performed.

Outcome:

There was a 374% increase in systemic exposure at steady state (30700 ng/mL vs. 146000 ng/mL) and 312% increase in maximum plasma concentration (3510 ng/mL vs. 14500 ng/mL) at 240 mg of basmisanil. There were no epileptiform discharges, EEG seizure activity and non-epileptiform clinically relevant abnormalities when basmisanil was co-administered with itraconazole.
